# Supplementary material for: The bacterial replication origin BUS promotes nucleobase capture
Source: Nat Commun. 2023 Dec 14;14:8339. doi: 10.1038/s41467-023-43823-w (PMC10721633; doi:10.1038/s41467-023-43823-w)
Supplement: Supplementary file 1 — Supplementary Information [file 41467_2023_43823_MOESM1_ESM.pdf]

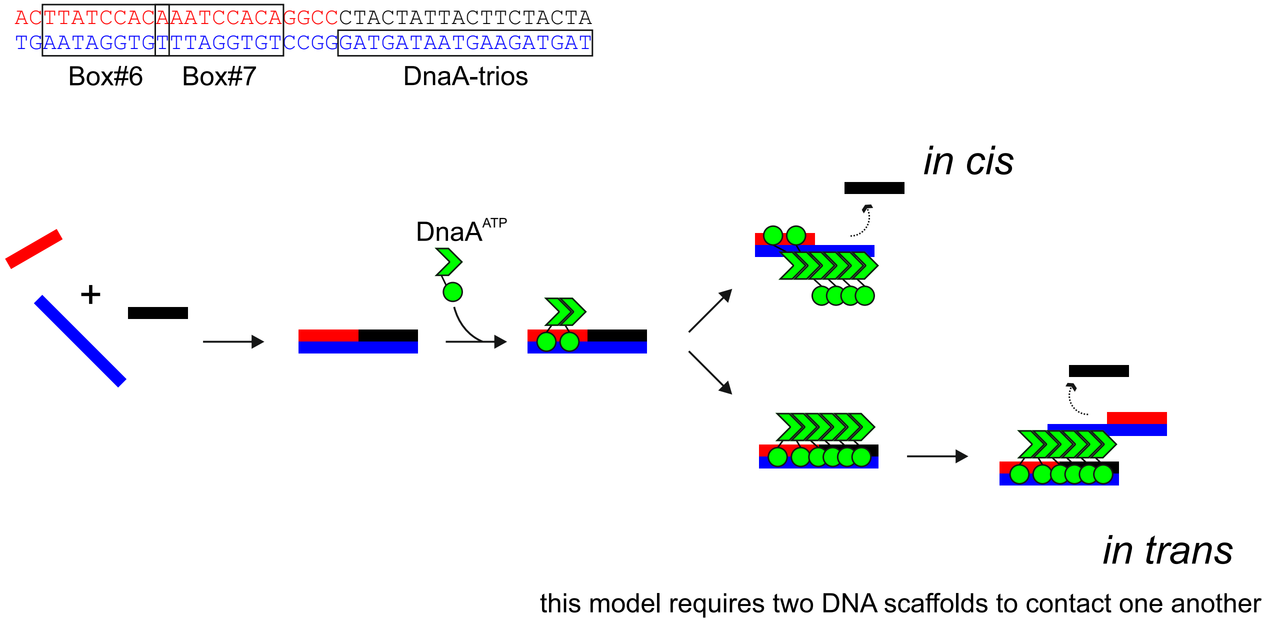

Supplementary Figure 1. Alternative models for BUS activity *in vitro.* DnaA-boxes are required to load DnaA onto DnaA-trios. The DNA scaffolds used to detect DnaA strand separation activity in solution are compatible with two models. In one, $DnaA^{ATP}$ is loaded from DnaA-boxes onto adjacent DnaA-trios (*in cis*). In another, an oligomer of $DnaA^{ATP}$ is nucleated from DnaA-boxes along the DNA scaffold, binding to the dsDNA using domain IV. This $DnaA^{ATP}$ oligomer can subsequently engage the DnaA-trios on a second DNA scaffold, binding to the ssDNA using domain III (*in trans*).

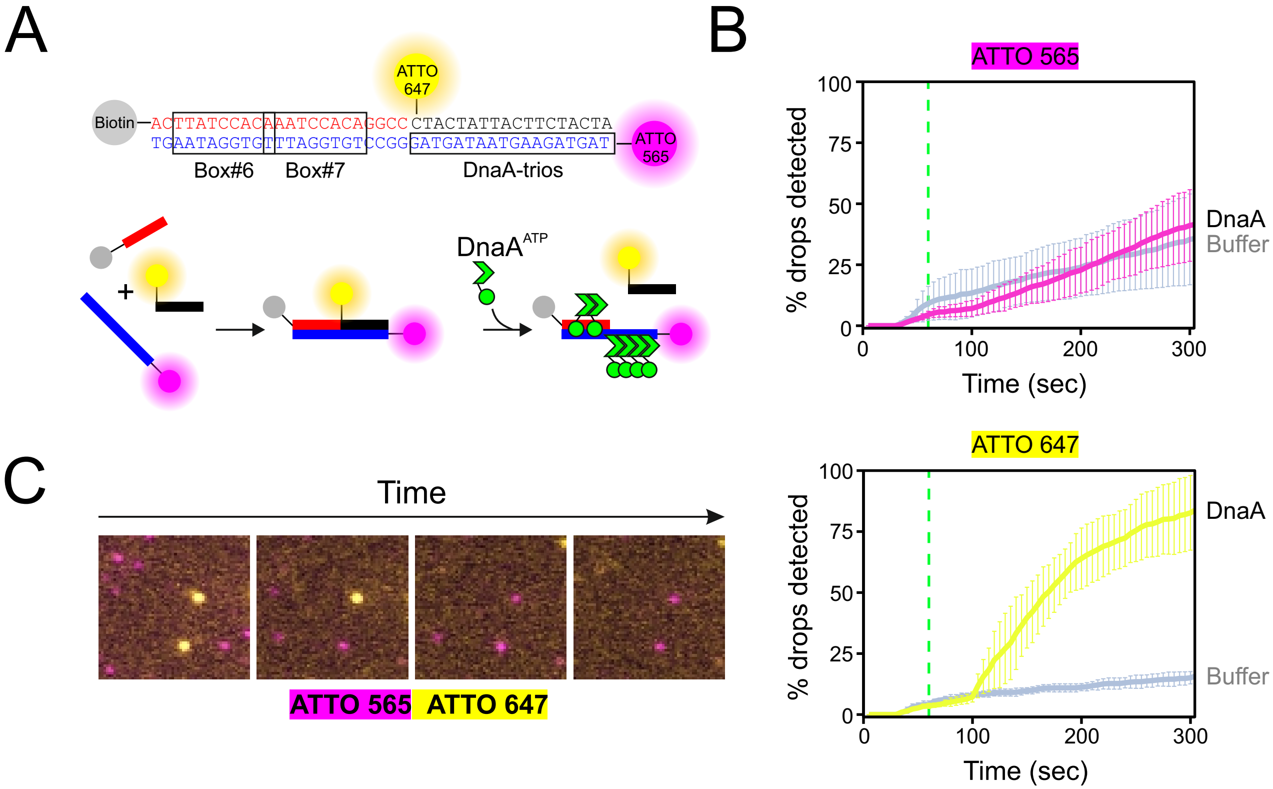

Supplementary Figure 2. A dual labelled DNA scaffold shows DnaA strand separation is specific. (A) Experimental design using a DNA scaffold containing two fluorescently labelled oligonucleotides. **(B)** Graph of colocalized fluorescent signals representing the percentage with decreased intensity over time ($ATTO^{565}$ pink, $ATTO^{647}$ yellow). Data shows the average and percentage standard deviation from three independent experiments (source data are provided as a Source Data file). **(C)** Example showing specific loss of $ATTO^{647}$ fluorescence (yellow), indicating a DnaA-dependent strand separation event.

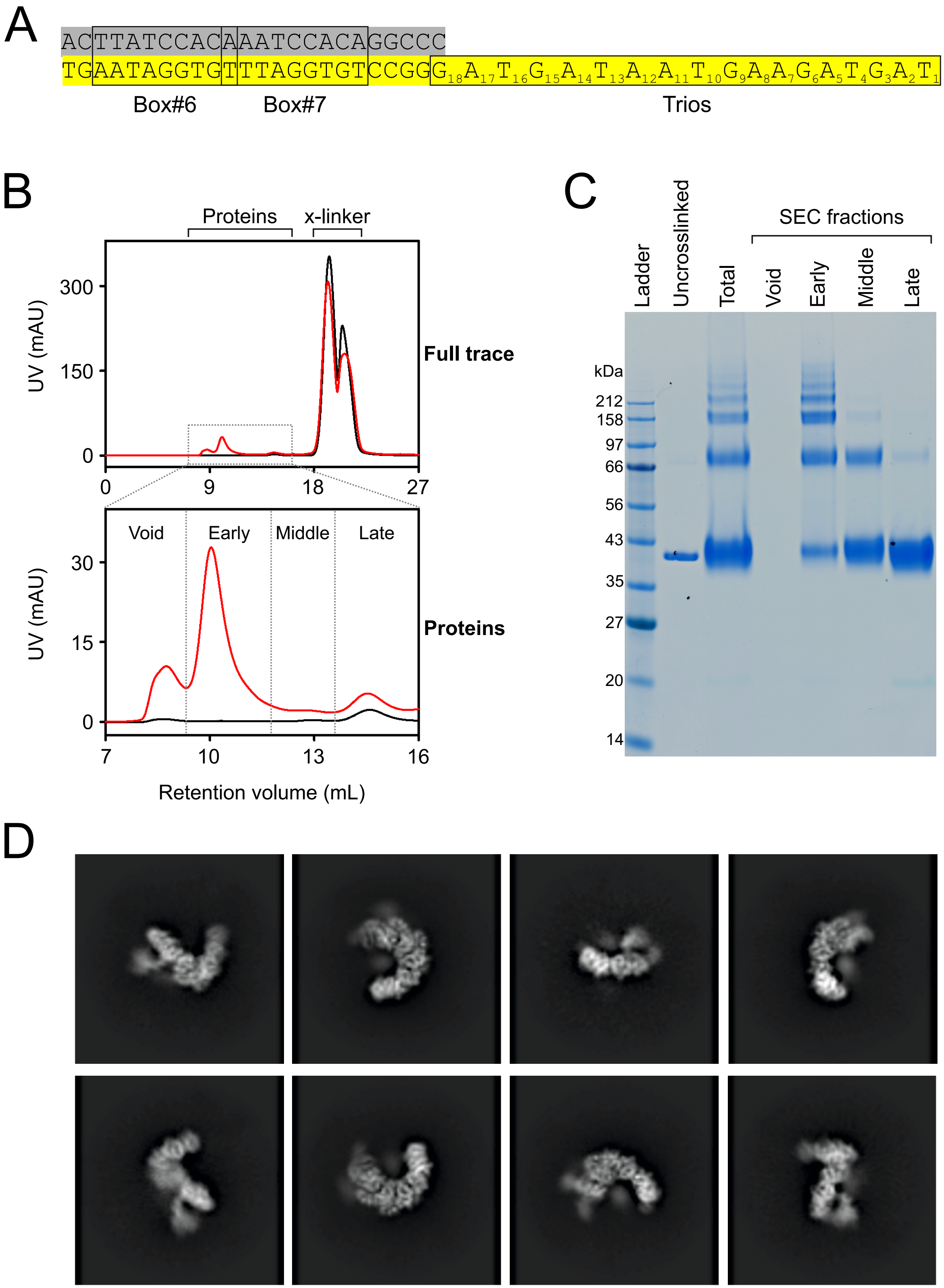
A
ACTTATCCACAAATCCACAGGCCC
TGAATAGGTGTTTAGGTGTCCGGG18A17T16G15A14T13A12A11T10G9A8A7G6A5T4G3A2T1
Box#6
Box#7
Trios
B
Proteins
x-linker
UV (mAU)
300
150
0
0
9
18
27
Full trace
Void
Early
Middle
Late
30
15
0
7
10
13
16
Proteins
Retention volume (mL)
C
Ladder
Uncrosslinked
Total
SEC fractions
Void
Early
Middle
Late
kDa
212
158
97
66
56
43
35
27
20
14
D

Supplementary Figure 3. Biochemistry and cryo-EM analysis of the BUS. (A) The DNA scaffold used for single particle cryo-EM structural studies. **(B)** Size exclusion chromatography to separate the BUS. **(C)** SDS-PAGE gel showing the glutaraldehyde cross-linked sample isolated for cryo-EM. **(D)** Representative 2D class averages of the BUS obtained using CryoSparc v3.1.2[37].

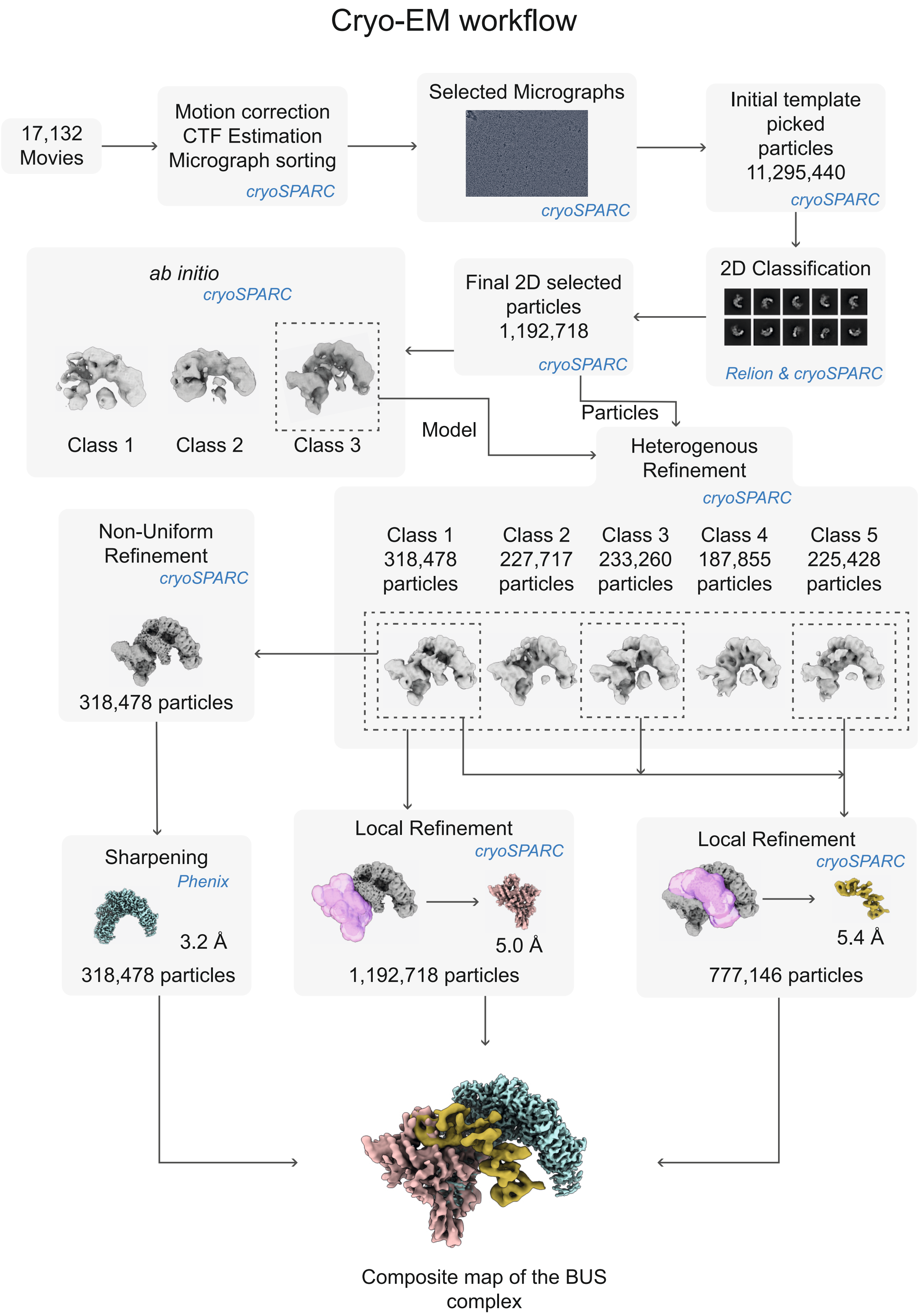

Cryo-EM workflow
17,132 Movies
Motion correction
CTF Estimation
Micrograph sorting
cryoSPARC
Selected Micrographs
cryoSPARC
Initial template picked particles 11,295,440
cryoSPARC
2D Classification
Relion & cryoSPARC
Final 2D selected particles 1,192,718
cryoSPARC
ab initio
cryoSPARC
Class 1
Class 2
Class 3
Model
Particles
Heterogenous Refinement
cryoSPARC
Class 1 318,478 particles
Class 2 227,717 particles
Class 3 233,260 particles
Class 4 187,855 particles
Class 5 225,428 particles
Non-Uniform Refinement
cryoSPARC
318,478 particles
Sharpening
Phenix
3.2 Å
318,478 particles
Local Refinement
cryoSPARC
5.0 Å
1,192,718 particles
Local Refinement
cryoSPARC
5.4 Å
777,146 particles
Composite map of the BUS complex

Supplementary Figure 4. Cryo-EM workflow. Schematic of the single particle cryo-EM workflow showing the classification, refinement and sharpening steps used to obtain a composite map of the BUS nucleoprotein complex.

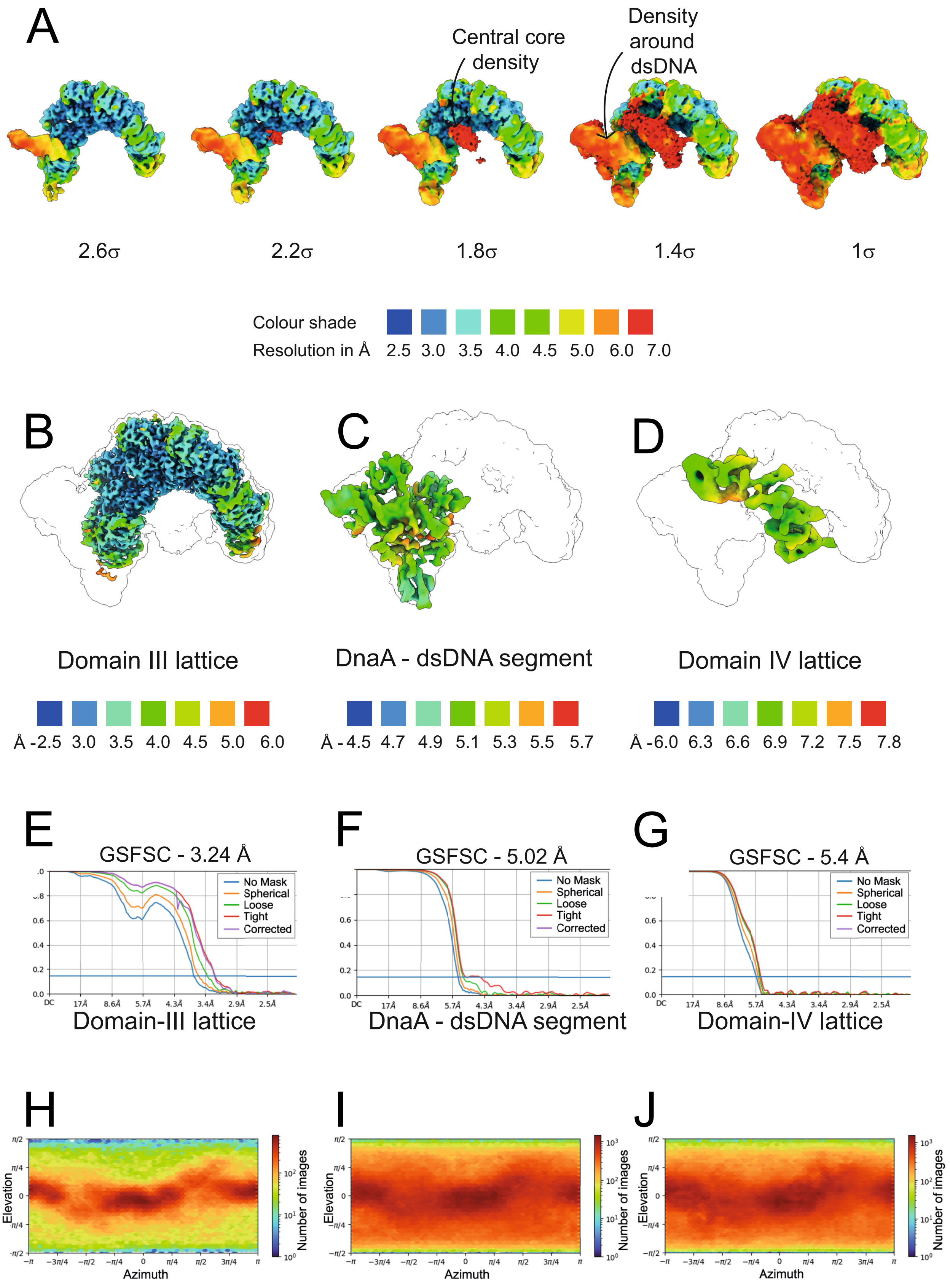
A
Central core density
Density around dsDNA
2.6σ
2.2σ
1.8σ
1.4σ
1σ
Colour shade
Resolution in Å 2.5 3.0 3.5 4.0 4.5 5.0 6.0 7.0
B
C
D
Domain III lattice
DnaA - dsDNA segment
Domain IV lattice
Å -2.5 3.0 3.5 4.0 4.5 5.0 6.0
Å - 4.5 4.7 4.9 5.1 5.3 5.5 5.7
Å -6.0 6.3 6.6 6.9 7.2 7.5 7.8
E
F
G
GSFSC - 3.24 Å
GSFSC - 5.02 Å
GSFSC - 5.4 Å
No Mask
Spherical
Loose
Tight
Corrected
Domain-III lattice
DnaA - dsDNA segment
Domain-IV lattice
H
I
J
Elevation
Azimuth
Number of images

Supplementary Figure 5. Cryo-EM maps used in this study. (A) The cryo-EM maps of the BUS coloured by local resolution with each map displayed at different σ levels. Additional density corresponding to the central core and protein binding double stranded DNA can be seen emerging at higher σ levels. **(B)** Cryo-EM map of the domain III lattice coloured by local resolution contoured at 0.6σ, overlayed on the overall BUS map contoured at 0.1σ. **(C)** Cryo-EM map of the region around the double stranded DNA coloured by local resolution contoured at 0.27σ, overlayed on the overall BUS map contoured at 0.1σ. **(D)** Cryo-EM map of the domain IV lattice coloured by local resolution contoured at 0.21σ, overlayed on the overall BUS map contoured at 0.1σ. **(E)** Fourier shell correlation of the cryo-EM map for the domain III lattice showing an average map resolution of 3.2 Å. **(F)** Fourier shell correlation of the cryo-EM map for the region around dsDNA showing an average map resolution of 5.0 Å. **(G)** Fourier shell correlation of cryo-EM map for the domain IV lattice map showing an average map resolution of 5.4 Å. **(H)** Orientation distribution of particles contributing for the cryo-EM map of the domain-III lattice. **(I)** Orientation distribution of particles contributing for the cryo-EM map of the region around dsDNA. **(J)** Orientation distribution of particles contributing for the cryo-EM map of the domain-IV lattice.

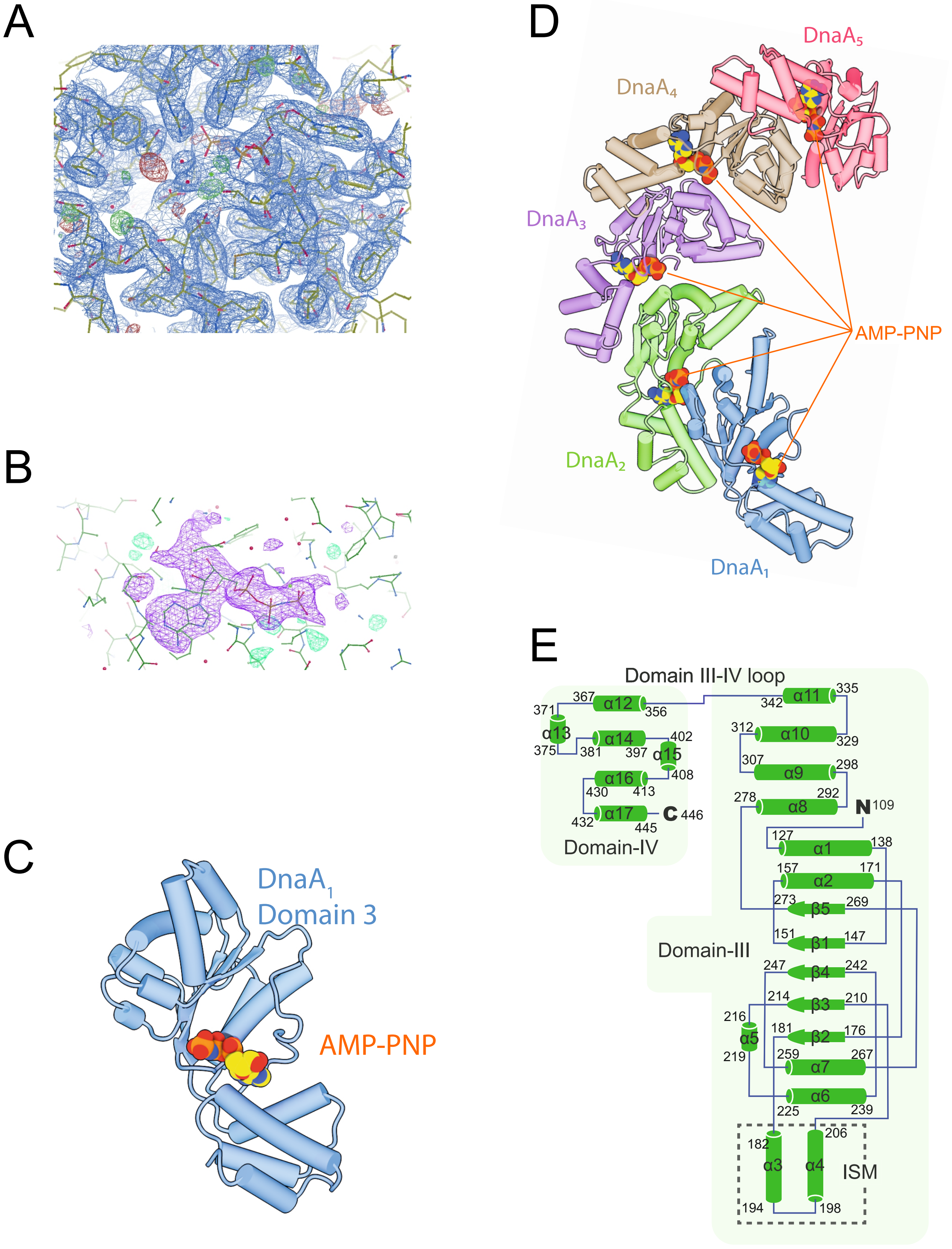
A
B
C
DnaA1
Domain 3
AMP-PNP
D
DnaA5
DnaA4
DnaA3
DnaA2
DnaA1
AMP-PNP
E
Domain III-IV loop
Domain-IV
Domain-III
ISM
α1
α2
α3
α4
α5
α6
α7
α8
α9
α10
α11
α12
α13
α14
α15
α16
α17
β1
β2
β3
β4
β5
N 109
C 446

Supplementary Figure 6. X-ray crystal structure of *B. subtilis* DnaA$^{106\text{-}346}$ determined at 2.38 Å. (A) Representative view of the electron density map (contour level is 1σ). **(B)** An omit map of the bound ligand (contour level is 3σ). **(C)** A monomer of DnaA$^{106\text{-}346}$ (domain III). The AMP-PNP bound to the protein is shown in spacefill. **(D)** In the unit cell, five DnaA monomers are organised in a right-handed helical oligomer. **(E)** A topology diagram of the DnaA domain III and IV showing the respective domains and secondary structure. The ISM is highlighted.

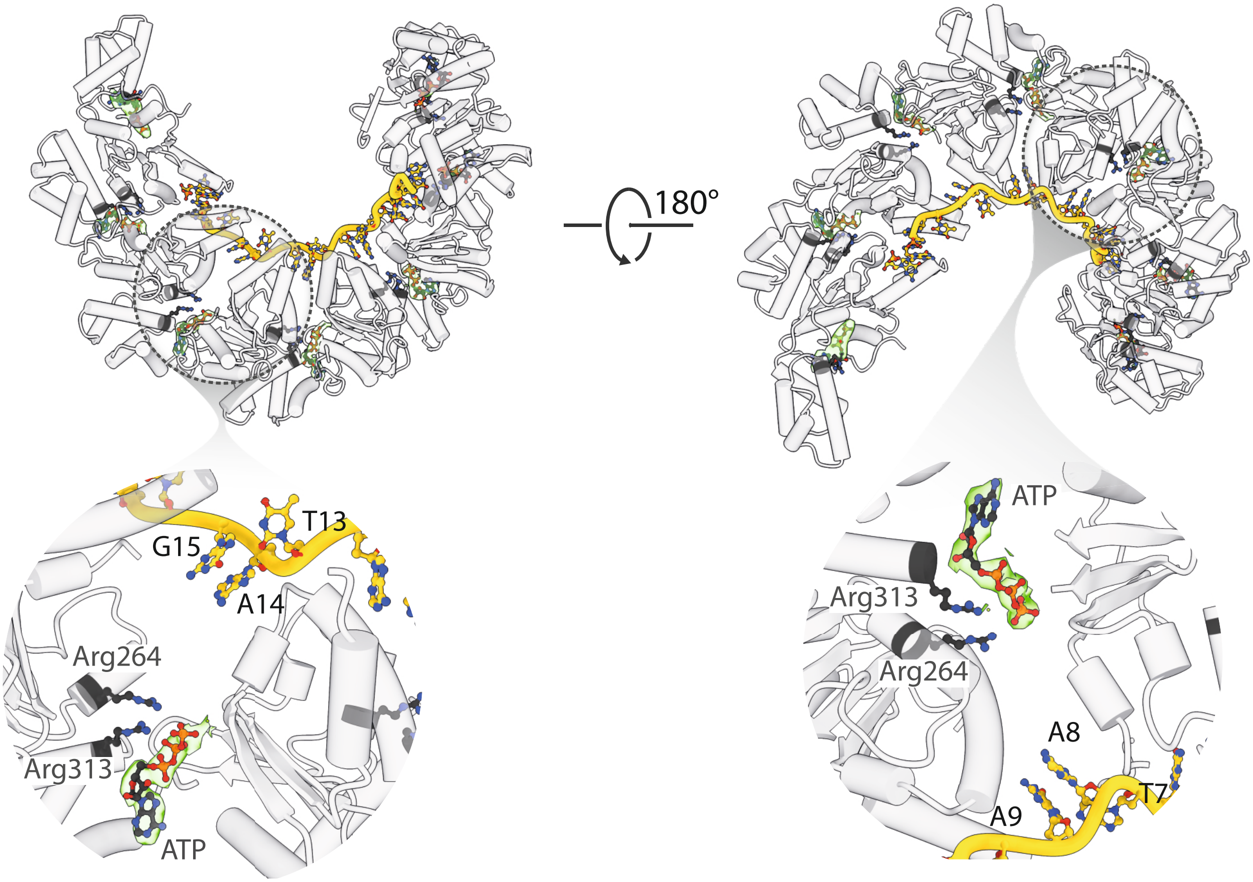

Supplementary Figure 7. Orientation of the DnaA oligomer on ssDNA. Model of the BUS complex showing domain III bound to ssDNA of the DNA scaffold. The ssDNA, ATP, and key arginine residues (Arg264 and Arg333) interacting with ATP are highlighted. The left inset shows the ATP (with extracted density contoured at 0.6σ) that is stabilized by Arg264 of $DnaA_2$ and Arg313 of $DnaA_3$. The DnaA-trio composed of T13, A14, and G15 that engages $DnaA_2$ and $DnaA_3$ is also shown. The right inset shows the ATP (with density) that is stabilized by Arg264 of $DnaA_4$ and Arg313 of $DnaA_5$. The DnaA-trio composed of T7, A8, and A9 that engages $DnaA_4$ and $DnaA_5$ is also shown.

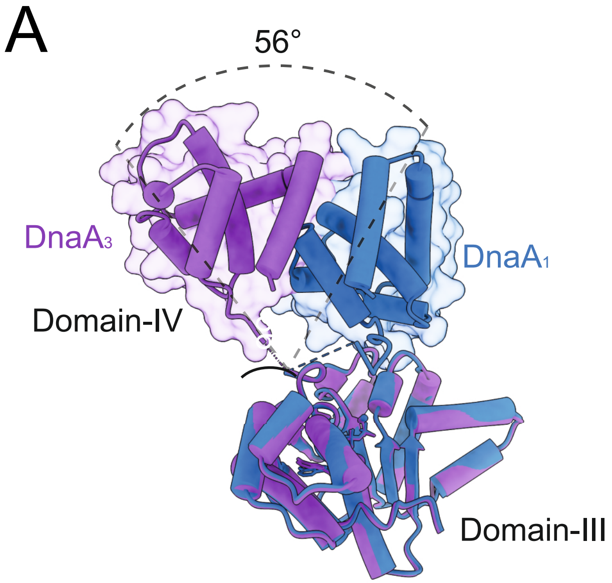

Supplementary Figure 8. Movement of Domain IV in DnaA proteins bound to DnaA-boxes. (A) Structural alignment of the $DnaA_1$ and $DnaA_3$ showing the domain IV of $DnaA_1$ and $DnaA_3$ are 56° apart relative to domain III. **(B)** Structural alignment of the $DnaA_2$ and $DnaA_3$ showing the domain IV of $DnaA_2$ and $DnaA_3$ are 37° apart while relative to domain III.

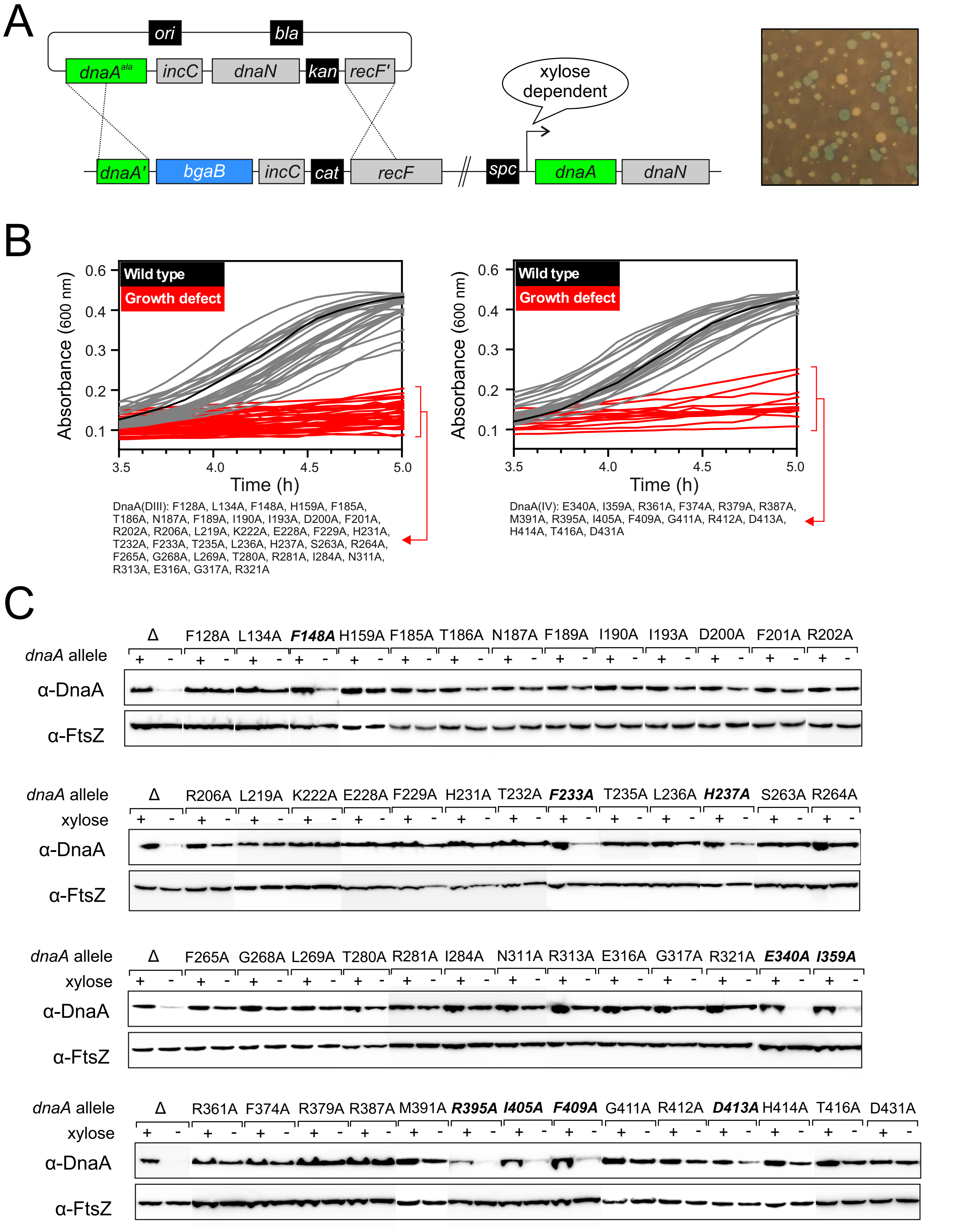

A
ori
bla
dnaA^ala
incC
dnaN
kan
recF'
dnaA'
bgaB
incC
cat
recF
spc
dnaA
dnaN
xylose dependent
B
Wild type
Growth defect
Absorbance (600 nm)
0.6
0.4
0.3
0.2
0.1
3.5
4.0
4.5
5.0
Time (h)
DnaA(DIII): F128A, L134A, F148A, H159A, F185A, T186A, N187A, F189A, I190A, I193A, D200A, F201A, R202A, R206A, L219A, K222A, E228A, F229A, H231A, T232A, F233A, T235A, L236A, H237A, S263A, R264A, F265A, G268A, L269A, T280A, R281A, I284A, N311A, R313A, E316A, G317A, R321A
DnaA(IV): E340A, I359A, R361A, F374A, R379A, R387A, M391A, R395A, I405A, F409A, G411A, R412A, D413A, H414A, T416A, D431A
C
dnaA allele
Δ F128A L134A F148A H159A F185A T186A N187A F189A I190A I193A D200A F201A R202A
α-DnaA
α-FtsZ
dnaA allele
xylose
Δ R206A L219A K222A E228A F229A H231A T232A F233A T235A L236A H237A S263A R264A
α-DnaA
α-FtsZ
dnaA allele
xylose
Δ F265A G268A L269A T280A R281A I284A N311A R313A E316A G317A R321A E340A I359A
α-DnaA
α-FtsZ
dnaA allele
xylose
Δ R361A F374A R379A R387A M391A R395A I405A F409A G411A R412A D413A H414A T416A D431A
α-DnaA
α-FtsZ

Supplementary Figure 9. Identification of essential residues in *B. subtilis* DnaA. (A) Method for constructing the *dnaA* alanine substitution library. An integration vector (pCW23) carrying individual *dnaA* substitutions was used for allelic replacement of the native *dnaA* gene (using the recipient strain CW199). Selection of white colonies following transformation of *dnaA* substitutions is performed in the presence of kanamycin, the chromogenic substrate X-gal and xylose (to express DnaA from the ectopic locus). **(B)** Plate reader growth assays of DnaA variants in the absence of xylose. The black line shows the growth profile of cells harbouring wild-type *dnaA*, whereas the lines coloured red show lethal substitutions (lines coloured grey grew similar to wild-type). Lists of the DnaA variants that revealed a lethal phenotype are provided below each panel. The wild-type control is CW380. Strains of the alanine substitution library are listed in Supplementary Table 1. **(C)** Immunoblotting reveals that some lethal alanine substitutions in the endogenous copy of DnaA were not well expressed (italicized and bold). The tubulin homolog FtsZ was used as a standard. The strain lacking *dnaA* at the native locus is FDS257. Multiple images have been spliced together to create the composite panel.

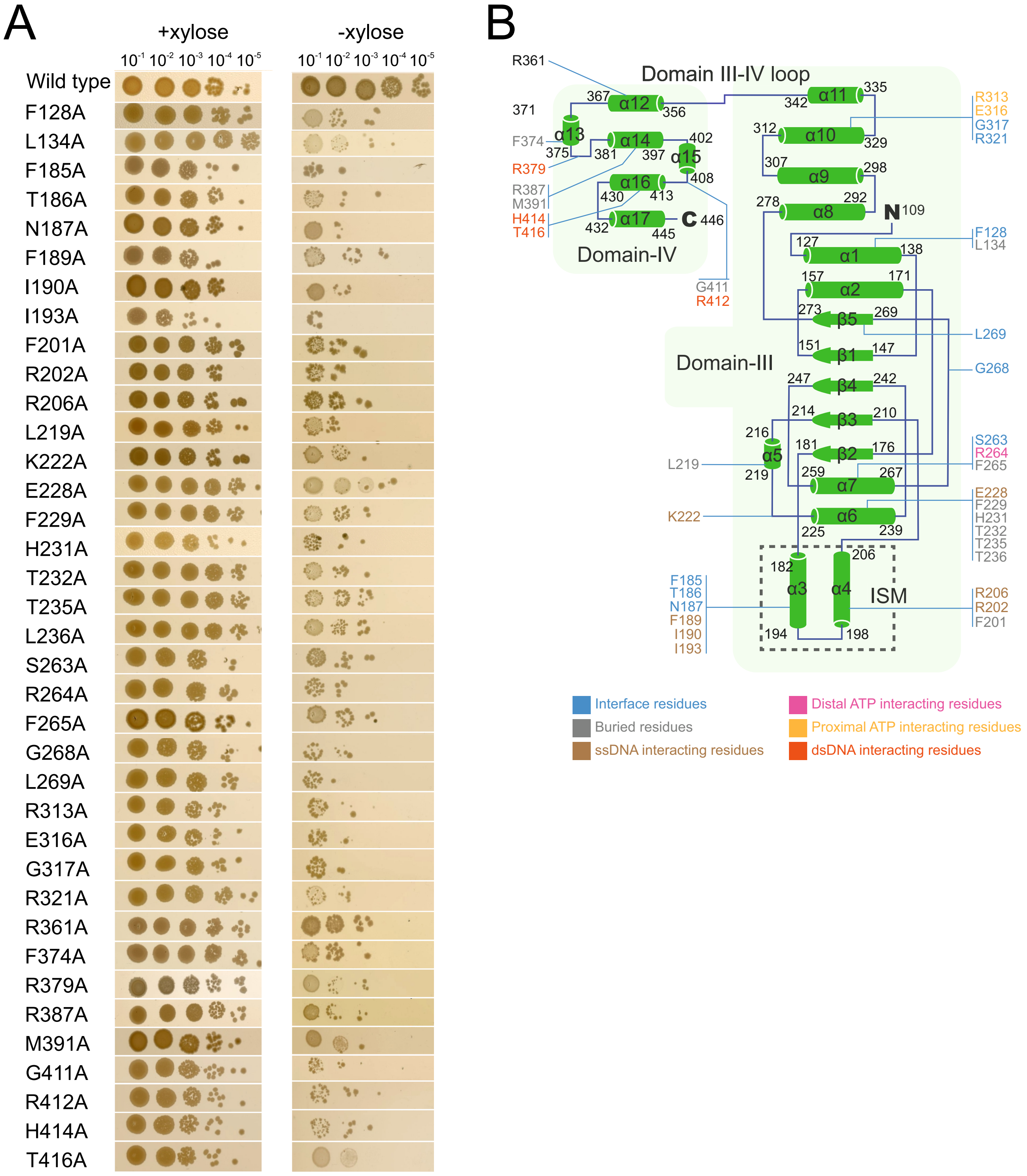

Supplementary Figure 10. Confirmation and mapping of essential DnaA residues. (A) Spot titre assays of lethal alanine substitutions that are expressed *in vivo*. The wild-type control is CW233 and the parent strain of all DnaA mutants is CW199. **(B)** Topology diagram of DnaA protein with the locations of lethal alanine substitutions indicated. The proposed activity of each essential residue is also indicated.

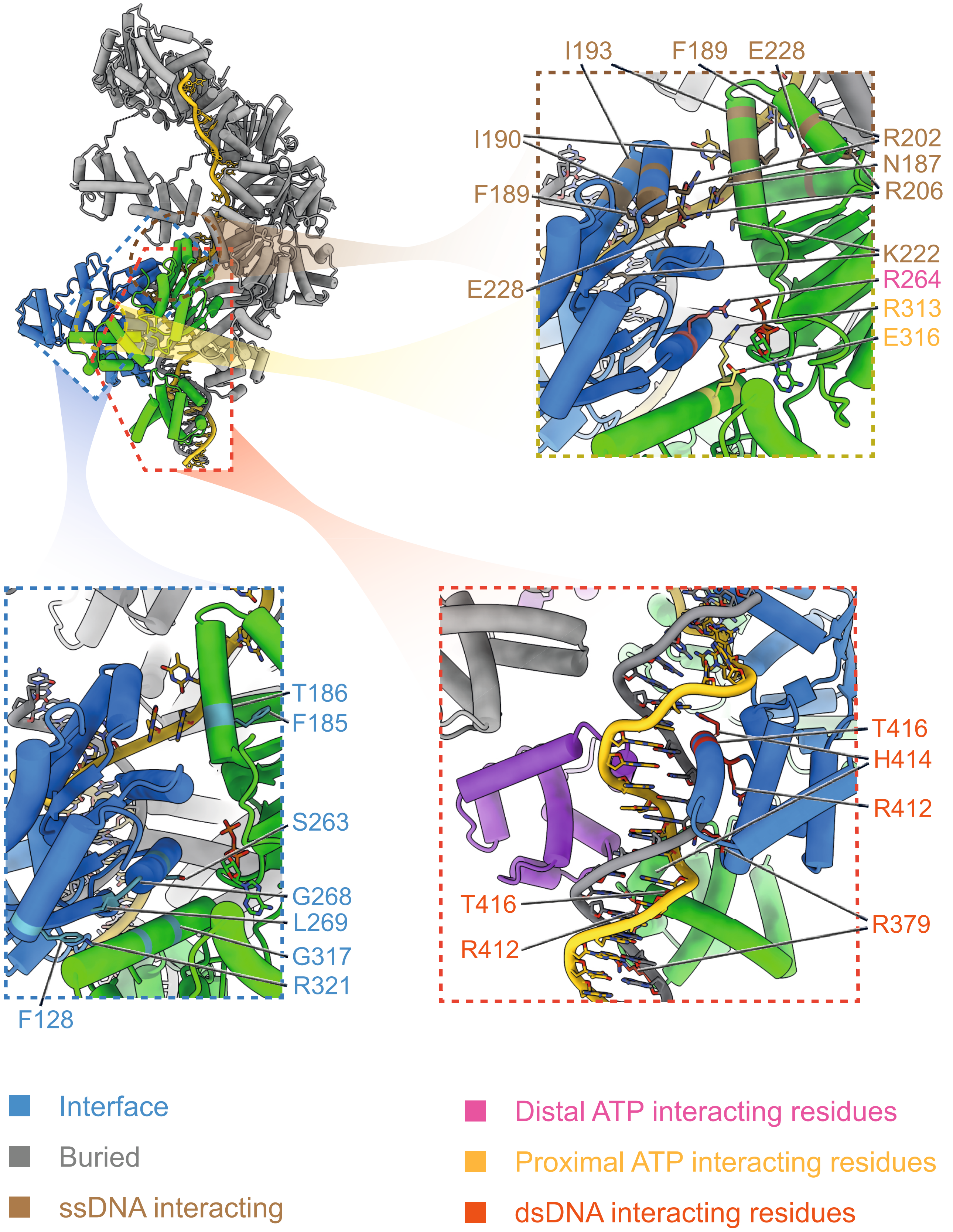
I193
F189
E228
I190
F189
E228
R202
N187
R206
K222
R264
R313
E316
T186
F185
S263
G268
L269
G317
R321
F128
T416
H414
R412
T416
R412
R379
Interface
Buried
ssDNA interacting
Distal ATP interacting residues
Proximal ATP interacting residues
dsDNA interacting residues

Supplementary Figure 11. Mapping essential residues onto the model of DnaA. Top left, the overall BUS complex model shown in grey, with $DnaA_1$ and $DnaA_2$ highlighted in blue and green, respectively. Top right inset shows the interface between $DnaA_1$ and $DnaA_2$ with the ssDNA interacting residues shown in brown and proximal ATP interacting residues shown in yellow. Bottom left inset show the interface between $DnaA_1$ and $DnaA_2$ highlighting key residues required for oligomerisation. Bottom right inset shows the domain IV of $DnaA_1$, $DnaA_2$ and $DnaA_3$ with the residues involved in dsDNA interaction highlighted in orange.

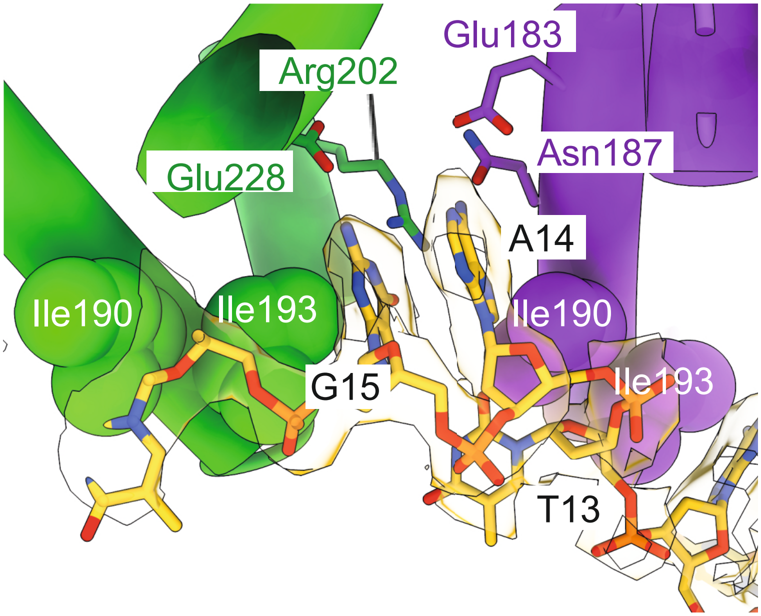

Supplementary Figure 12. Essential hydrophobic residues flank flipped bases. Hydrophobic side chains within the ISM (residues Ile190 and Ile193) are inserted between the two flipped bases (G18, A17) and the non-flipped base (T16). The extracted density around the bases are contoured at 0.6σ.

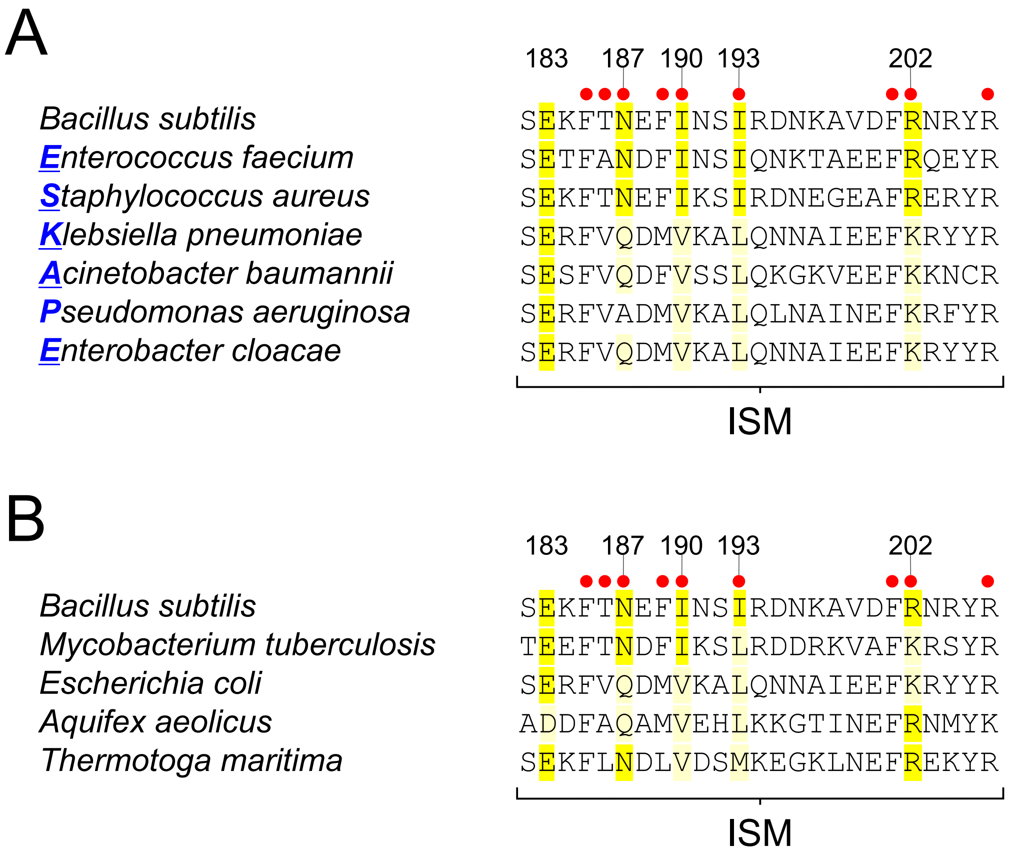

Supplementary Figure 13. Primary domain organisation and sequence conservation of DnaA proteins. Alignments of residues around the DnaA ISM involved in binding DnaA-trios (numbering refers to *B. subtilis*). Red dots indicate that alanine substitution in *B. subtilis* is lethal. **(A)** DnaA homologs from the multidrug resistance ESKAPE pathogens. **(B)** DnaA homologs from commonly used model organisms.

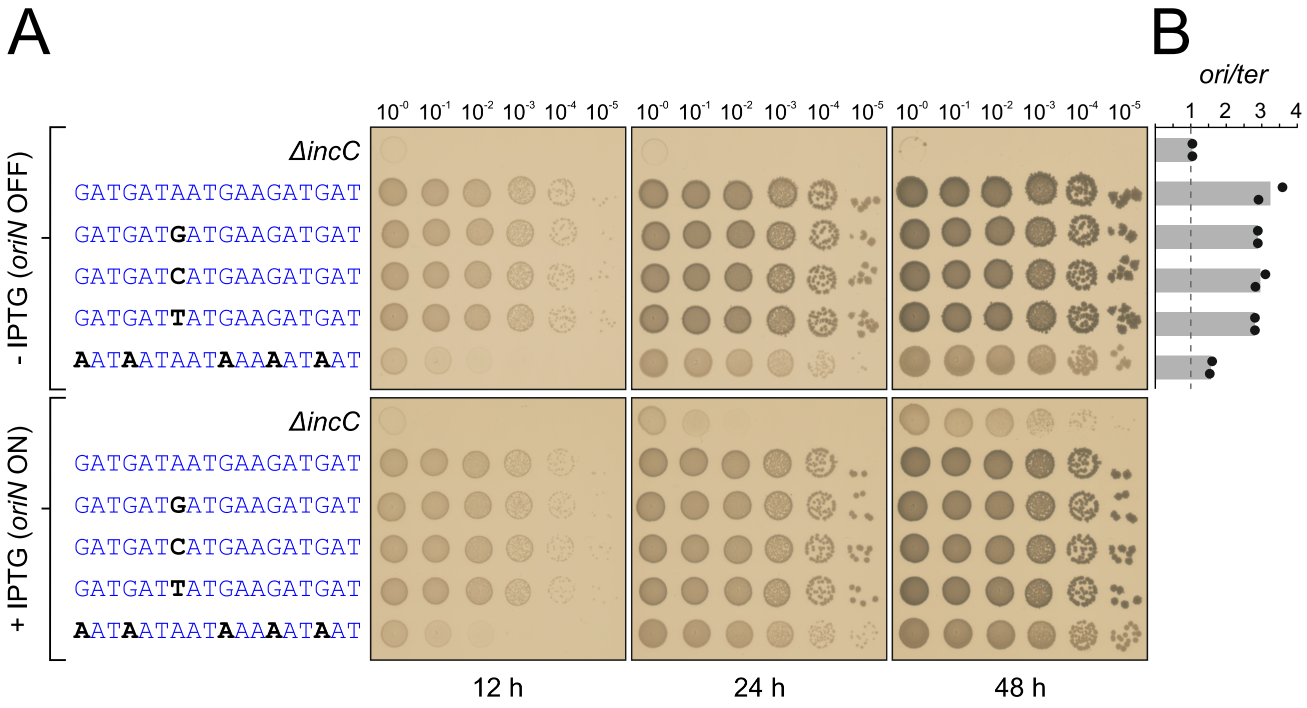

Supplementary Figure 14. The dinucleotide 3′-GA-5′ is preferred to 3′-AA-5′ within DnaA-trios. (A) Spot titre analysis with strains that have replaced DnaA-trio$_3$ 3′-AAT-5′ with either 3′-GAT-5′, 3′-CAT-5′ or 3′-TAT-5′ and a strain harbouring adenine in the first position of each DnaA-trio. The presence or absence of IPTG indicates the induction state of the *repN/oriN* system. Wild-type (FS682), Δ*incC* (FS404), DnaA-trio$_3$ first position: A→G (FDS1029), A→C (FDS1031), A→T (FDS1035), all DnaA-trios first position A (FDS1037). (B) Marker frequency analysis of strains shown in panel A. Data shows the average and individual data points from two independent experiments (source data are provided as a Source Data file).

Supplementary Table 1.
X-ray data collection and refinement statistics

	DnaA domain III
**Data collection**	
Space group	P 32
Cell dimensions	
a, *b*, *c* (Å)	81.58, 81.58, 223.20
α, β, γ (°)	90, 90, 120
Resolution (Å)	74.40-2.38 (2.52-2.38)
R_{merge}	0.18 (1.46)
$I / \sigma I$	10.1 (1.5)
Completeness (%)	85.3 (27.2)
Redundancy	9.7 (6.7)
**Refinement**	
Resolution (Å)	74.40-2.38
No. reflections	54023
R_{work} / R_{free}	0.19/0.24
No. atoms	9656
Protein	9365
Ligand/ion	169
Water	122
B-factors	
Protein	50.45
Ligand/ion	40.03
Water	33.96
R.m.s. deviations	
Bond lengths (Å)	0.72
Bond angles (°)	0.86

Supplementary Table 2.
Cryo-EM data collection, processing, and model refinement statistics

**Data collection and processing**			
Microscope	Titan Krios		
No of Movies	17132		
Camera	K3		
Voltage (kV)	300		
Electron exposure (e^{-} $Å^{-2}$)	48.3		
Pixel size (Å)	1.072		
Defocus range (µm)	-0.7 to -3.0		
Symmetry imposed	C1		
	**Domain III lattice**	**dsDNA region**	**Domain IV lattice**
Initial particle images	4,970,535	4,970,535	4,970,535
Final particle images	318,478	1,192,718	777,146
FSC threshold	0.143	0.143	0.143
Map resolution (Å)	3.2	5.0	5.4
Map B factor	-137	-200	-100
EMDB codes	16230	16231	16256
**Refinement and model validation**			
Model vs data CC (mask)	0.70	0.49	0.62
Clash score	19.42		
Molprobity score	1.96		
Bond length rmsd (Å)	0.005		
Bond angle rmsd (Å)	0.925		
Poor rotamers (%)	0.54		
Ramachandran			
Favoured (%)	96.98		
Allowed (%)	3.02		
Outlier (%)	0		
PDB code	8BTG		
