# Supplementary material for: The bacterial replication origin BUS promotes nucleobase capture
Source: Nat Commun. 2023 Dec 14;14:8339. doi: 10.1038/s41467-023-43823-w (PMC10721633; doi:10.1038/s41467-023-43823-w)
Supplement: Supplementary file 3 — Description of Additional Supplementary Files [file 41467_2023_43823_MOESM3_ESM.pdf]

Description of Additional Supplementary Files

Title: Supplementary Data 1.
Description: Lists of strains, plasmids, and oligonucleotides used in this study. The measured melting temperature of DNA scaffolds are indicated.

Title: Supplementary Movie 1.
Description: Composite cryo-EM map and complete model of the BUS complex. DnaA protomers are individually coloured and labelled. The ATP molecules at the interface of two adjacent protomers are shown as spheres.

Title: Supplementary Movie 2.
Description: Model of the $DnaA_1$ and $DnaA_2$ engaging with dsDNA (Box#6 and Box#7) and $DnaA_3$ engaging with posterior side of the dsDNA as support to stabilize the Domain-IV lattice.

Title: Supplementary Movie 3.
Description: Model of DnaA-trio#2 bound to the dinucleotide binding pocket formed by $DnaA_2$ and $DnaA_3$ and showing the hydrogen bond interaction it makes with the protein side chains. The bases are labelled.
