# Supplementary material for: The bacterial replication origin BUS promotes nucleobase capture
Source: Nat Commun. 2023 Dec 14;14:8339. doi: 10.1038/s41467-023-43823-w (PMC10721633; doi:10.1038/s41467-023-43823-w)
Supplement: Supplementary file 8 — Reporting Summary [file 41467_2023_43823_MOESM8_ESM.pdf]

nature portfolio

Corresponding author(s): Heath Murray
Last updated by author(s): Sep 15, 2023

Reporting Summary

Nature Portfolio wishes to improve the reproducibility of the work that we publish. This form provides structure for consistency and transparency in reporting. For further information on Nature Portfolio policies, see our Editorial Policies and the Editorial Policy Checklist.

Statistics

For all statistical analyses, confirm that the following items are present in the figure legend, table legend, main text, or Methods section.

n/a	Confirmed	
☐	☒	The exact sample size (*n*) for each experimental group/condition, given as a discrete number and unit of measurement
☐	☒	A statement on whether measurements were taken from distinct samples or whether the same sample was measured repeatedly
☒	☐	The statistical test(s) used AND whether they are one- or two-sided<br>*Only common tests should be described solely by name; describe more complex techniques in the Methods section.*
☒	☐	A description of all covariates tested
☒	☐	A description of any assumptions or corrections, such as tests of normality and adjustment for multiple comparisons
☐	☒	A full description of the statistical parameters including central tendency (e.g. means) or other basic estimates (e.g. regression coefficient) AND variation (e.g. standard deviation) or associated estimates of uncertainty (e.g. confidence intervals)
☒	☐	For null hypothesis testing, the test statistic (e.g. *F*, *t*, *r*) with confidence intervals, effect sizes, degrees of freedom and *P* value noted<br>*Give P values as exact values whenever suitable.*
☒	☐	For Bayesian analysis, information on the choice of priors and Markov chain Monte Carlo settings
☒	☐	For hierarchical and complex designs, identification of the appropriate level for tests and full reporting of outcomes
☒	☐	Estimates of effect sizes (e.g. Cohen's *d*, Pearson's *r*), indicating how they were calculated

Our web collection on statistics for biologists contains articles on many of the points above.

Software and code

Policy information about availability of computer code

Data collection	Nikon NIS Elements software (v5.42.01) was used for microscope control and image acquisition.<br>Amersham Typhoon Software v3.0.0.2 was used for laser scanning.<br>Rotor-Gene Q Software v2.3.5 was used for qPCR.<br>BMG Clariostar Software (v5.20) was used for fluorescence detection in a plate reader.<br>Magellan software (v7.2) was used for absorbance detection in a plate reader.
Data analysis	AutoPROC (v1.0.5 20211020), STARANISO (v2.3.79), Phaser (v2.8.3), Consurf (April 2021), Refmac (5.8.0267).<br>Coot (v8.9.2), Phenix (1.20.1), Molprobity (v4.5), Phyre (v2).<br>CryoSPARC (v3.1.0), Relion (v4.0), EPU (FEI) (v3.1), MotionCor2 (v1.4.7).<br>Fiji (ImageJ 1.53c), SigmaPlot (v14).<br>A fully annotated ImageJ Macro describing fluorescence microscopy data analysis is available on GitHub (https://github.com/GMerces/DisappearingSpots) [DOI 10.5281/zenodo.8363454]

For manuscripts utilizing custom algorithms or software that are central to the research but not yet described in published literature, software must be made available to editors and reviewers. We strongly encourage code deposition in a community repository (e.g. GitHub). See the Nature Portfolio guidelines for submitting code & software for further information.

Data

Policy information about availability of data

All manuscripts must include a data availability statement. This statement should provide the following information, where applicable:

- Accession codes, unique identifiers, or web links for publicly available datasets
- A description of any restrictions on data availability
- For clinical datasets or third party data, please ensure that the statement adheres to our policy

Electron Microscopy Data Bank with accession codes EMD-16230 (domain III lattice), EMD-16231 (dsDNA region) and EMD-16256 (domain IV lattice).
Protein Data Bank with accession codes 8BTG (BUS complex).
Protein Data Bank with accession codes 8BV3 (DnaA domains III).

Human research participants

Policy information about studies involving human research participants and Sex and Gender in Research.

Reporting on sex and gender	N/A
Population characteristics	N/A
Recruitment	N/A
Ethics oversight	N/A

Note that full information on the approval of the study protocol must also be provided in the manuscript.

Field-specific reporting

Please select the one below that is the best fit for your research. If you are not sure, read the appropriate sections before making your selection.

☒ Life sciences ☐ Behavioural & social sciences ☐ Ecological, evolutionary & environmental sciences

For a reference copy of the document with all sections, see nature.com/documents/nr-reporting-summary-flat.pdf

Life sciences study design

All studies must disclose on these points even when the disclosure is negative.

Sample size	The sample size of the fluorescent DNA substrates for single molecule TIRF experiments was not predetermined. Here more than 100 fluorescent signals were quantified during each reaction.<br>The sample size of the micrographs and particles were not predetermined. A dataset of 17132 micrograph movies were collected and 16616 micrographs were selected and used for further processing. A final set of 1,192,718 particles were used to generate the initial 3D volume. All and selected subsets of the 1,192,718 particles were independently used to generate the three maps described in the study. The particle size was sufficient to achieve the reported resolutions of the three maps.
Data exclusions	No data was excluded from the single molecule TIRF experiments.<br>Exclusion of the cryo-EM micrographs were done based on quality of the image and ice, evaluated using the CTF information of the micrographs. Particle selection was done based on iterative 2D classification and heterogenous refinement with criteria based on the resulting 2D class average and 3D maps.
Replication	For single molecule TIRF experiments and bulk fluorescence assays, three independent repeats were performed to generate an average and determine the standard deviation. For marker frequency analysis, two independent experiments were performed.<br>All attempts at replication were successful for the cryo-EM studies. The data was processed with a different software (Relion-4.0) and similar results were obtained.
Randomization	During single particle data processing, the data was randomly split into two sets and processed the same way to calculate the Fourier Shell correlation (FSC) coefficient, in accordance with Gold Standard Methods. This randomization is embedded within the software programs used in the study.
Blinding	N/A

Reporting for specific materials, systems and methods

We require information from authors about some types of materials, experimental systems and methods used in many studies. Here, indicate whether each material, system or method listed is relevant to your study. If you are not sure if a list item applies to your research, read the appropriate section before selecting a response.

Materials & experimental systems

n/a	Involved in the study
☐	☒ Antibodies
☒	☐ Eukaryotic cell lines
☒	☐ Palaeontology and archaeology
☒	☐ Animals and other organisms
☒	☐ Clinical data
☒	☐ Dual use research of concern

Methods

n/a	Involved in the study
☒	☐ ChIP-seq
☒	☐ Flow cytometry
☒	☐ MRI-based neuroimaging

Antibodies

Antibodies used	Custom polyclonal antobodies were raised in rabbit against either recombinant DnaA or FtsZ from Bacillus subtilis.
Validation	Antibody specificity has been confirmed by performing immunoblots using strains lacking either DnaA (doi:10.15252/embj.2019101649) or FtsZ (doi:10.1038/nature07742).
