# Supplementary material for: The bacterial replication origin BUS promotes nucleobase capture
Source: Nat Commun. 2023 Dec 14;14:8339. doi: 10.1038/s41467-023-43823-w (PMC10721633; doi:10.1038/s41467-023-43823-w)
Supplement: Supplementary file 9 — Source Data [file 41467_2023_43823_MOESM9_ESM.zip › Source_Data_File_2_20231014.pptx]

## Slide 1
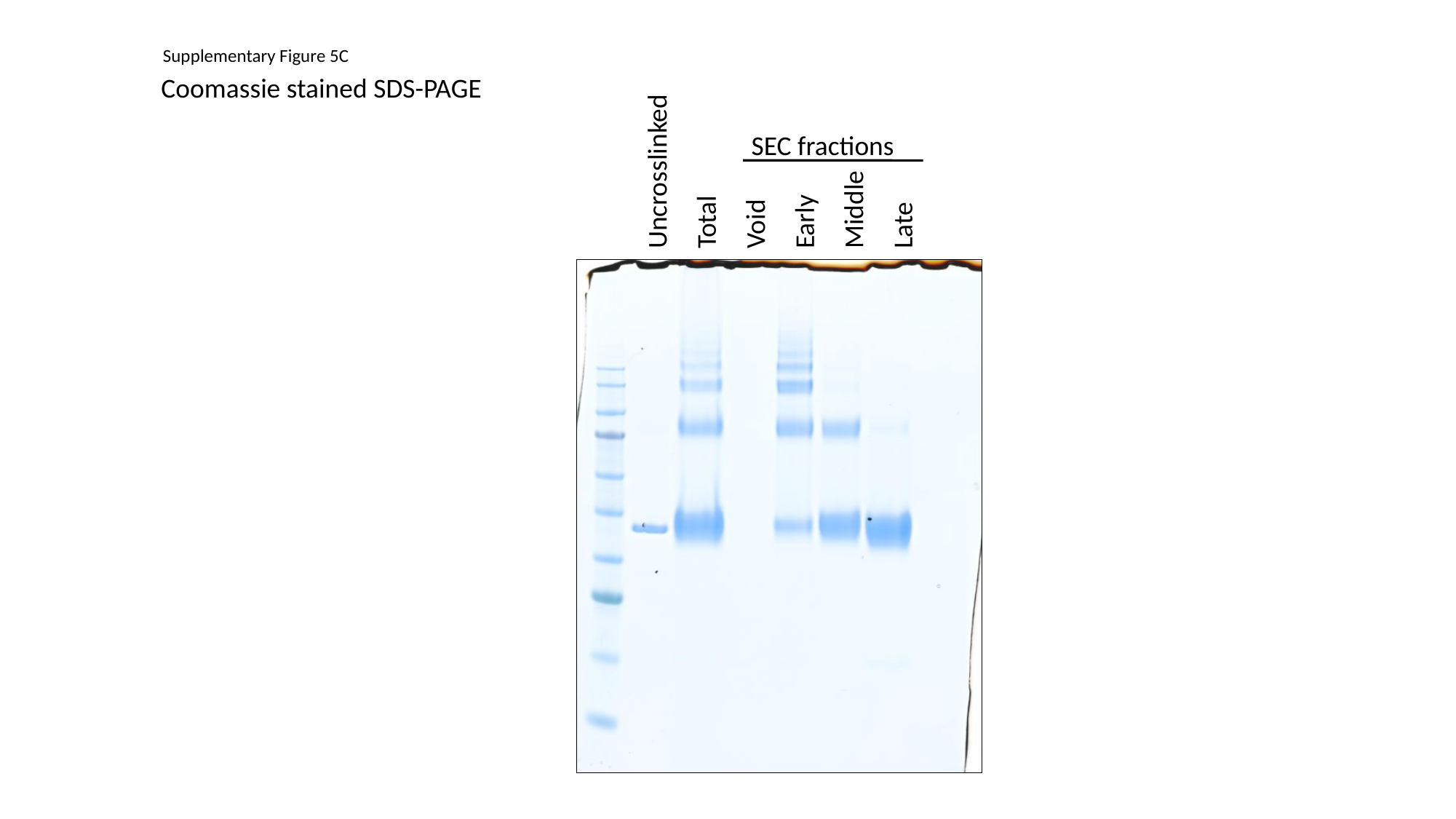

Uncrosslinked
Total
Void
Early
Middle
Late
Supplementary Figure 5C
Coomassie stained SDS-PAGE
SEC fractions

## Slide 2
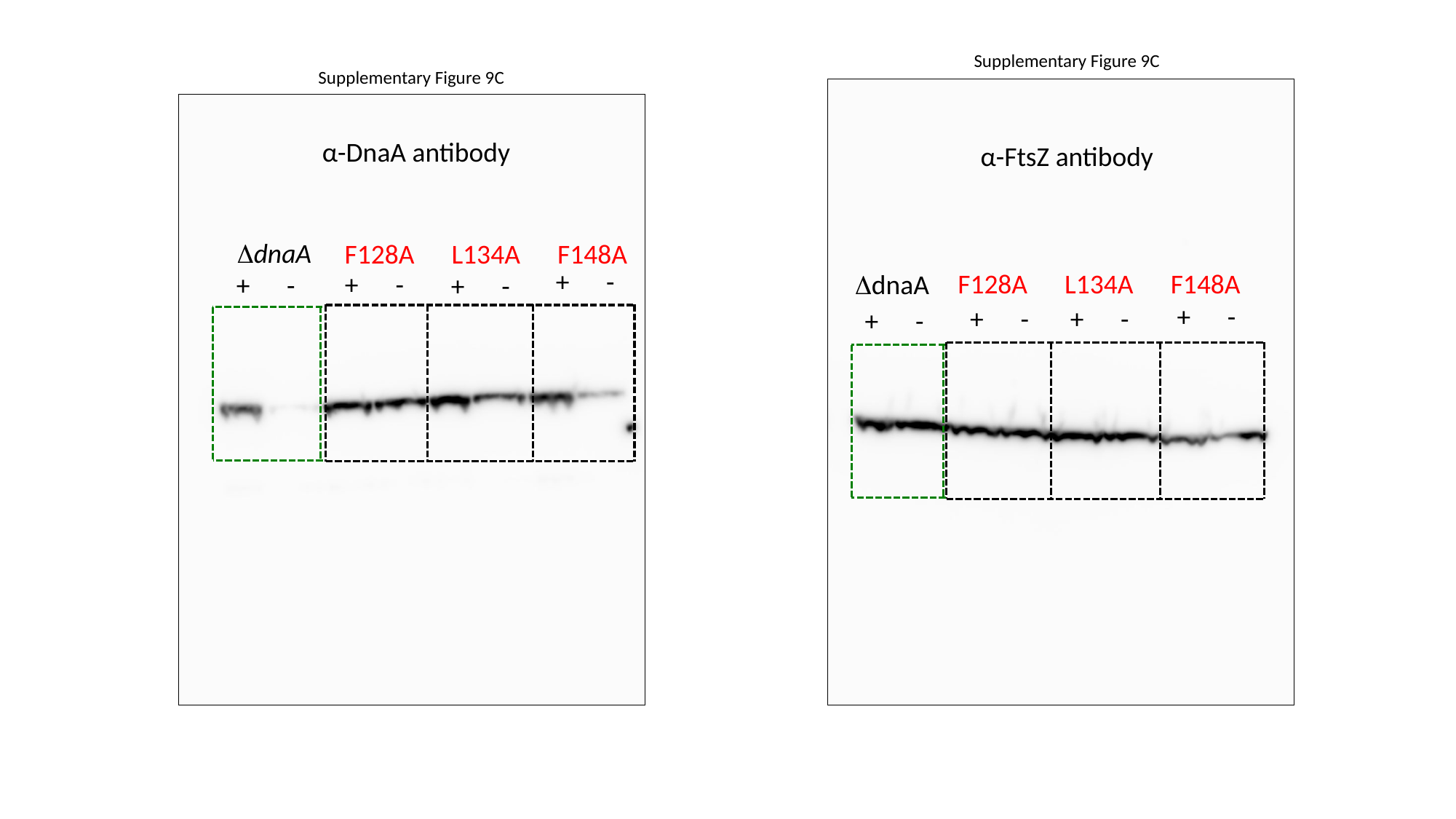

Supplementary Figure 9C
Supplementary Figure 9C
α-DnaA antibody
α-FtsZ antibody
DdnaA
F128A L134A F148A
+ -
F128A L134A F148A
+ -
DdnaA
+ -
+ -
+ -
+ -
+ -
+ -

## Slide 3
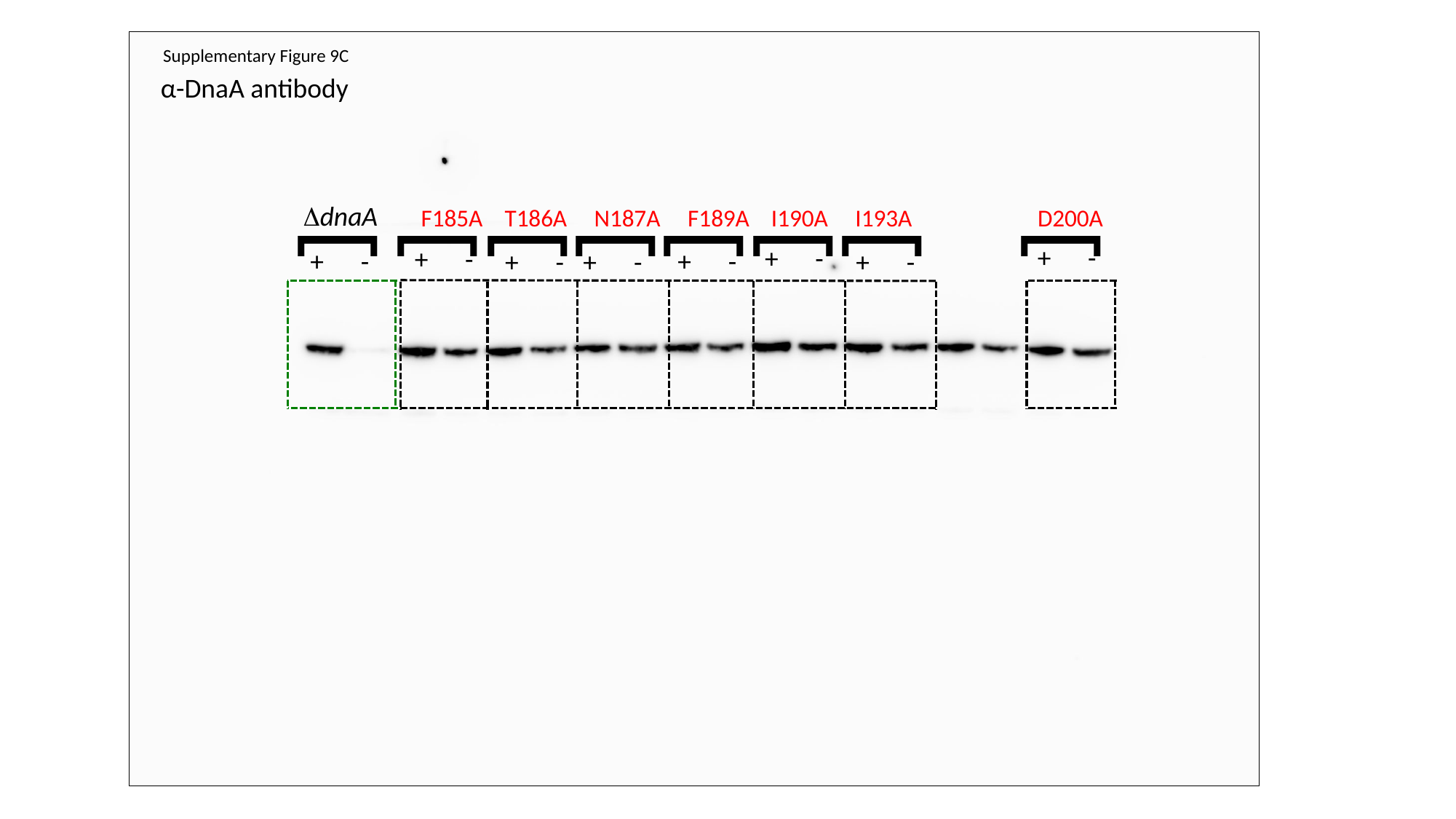

Supplementary Figure 9C
α-DnaA antibody
DdnaA
F185A T186A N187A F189A I190A I193A D200A
[
[
[
[
[
[
[
[
+ -
+ -
+ -
+ -
+ -
+ -
+ -
+ -

## Slide 4
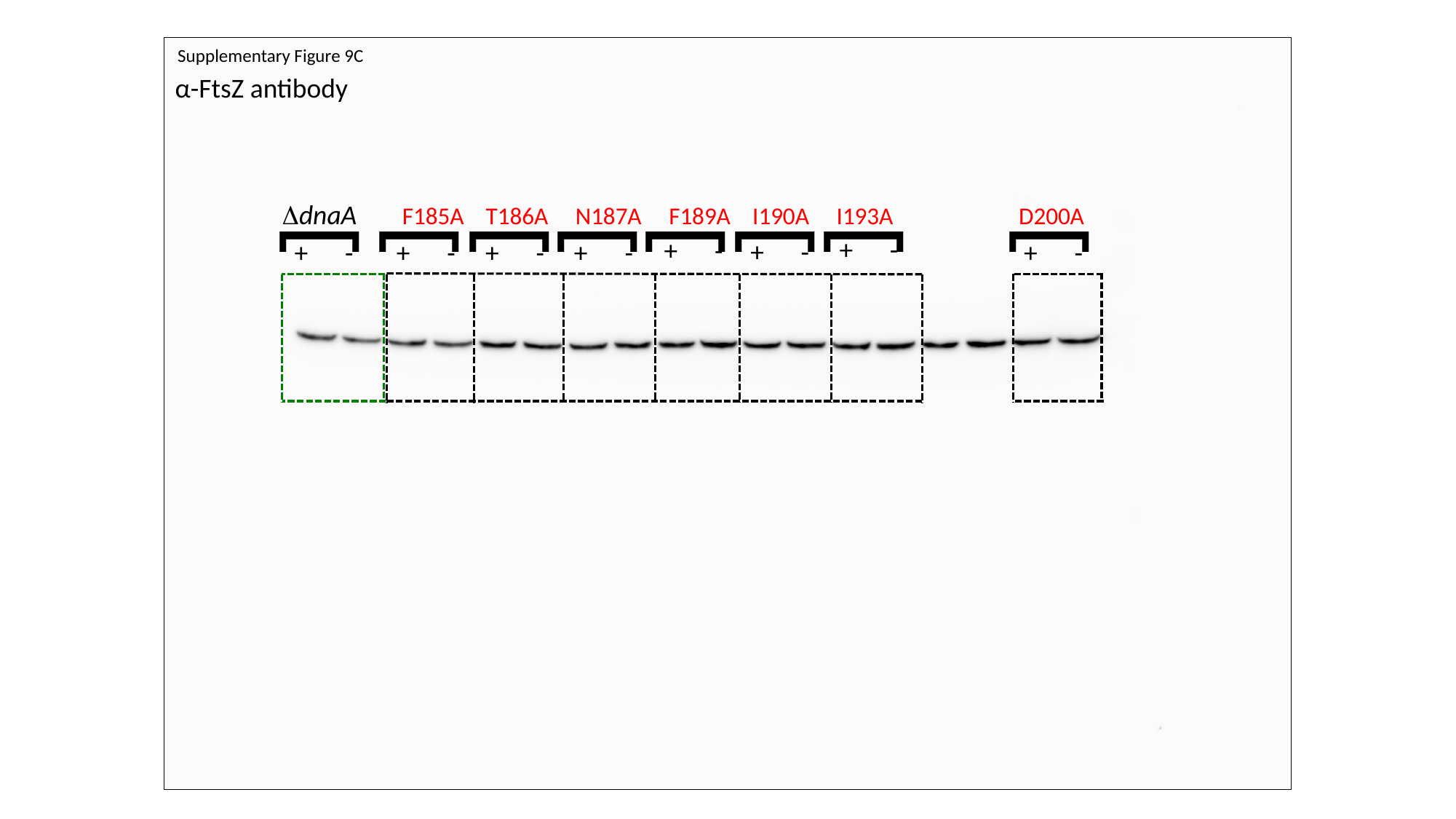

Supplementary Figure 9C
α-FtsZ antibody
[
[
[
[
[
[
[
DdnaA
F185A T186A N187A F189A I190A I193A D200A
[
+ -
+ -
+ -
+ -
+ -
+ -
+ -
+ -

## Slide 5
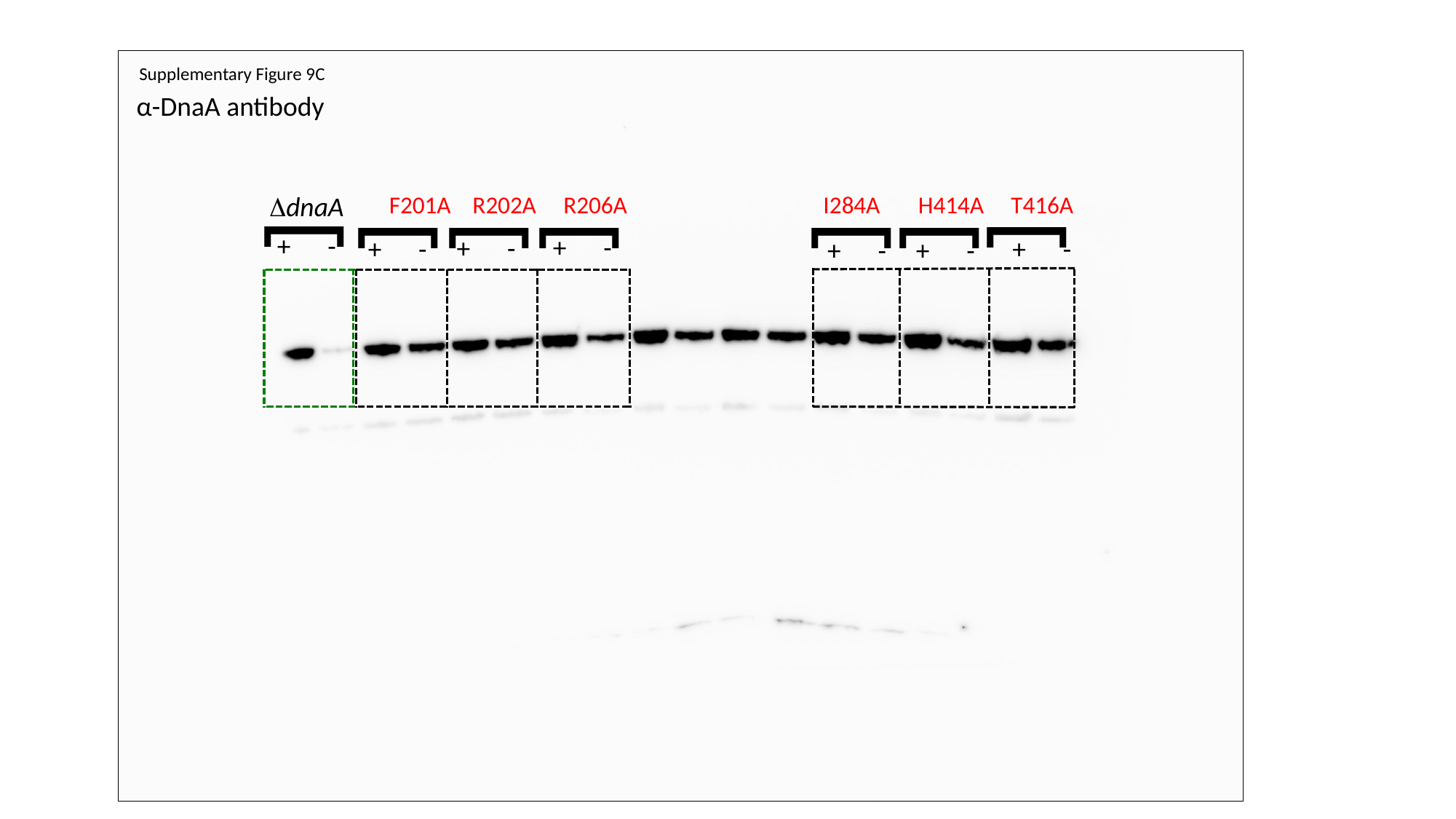

F201A R202A R206A I284A H414A T416A
Supplementary Figure 9C
α-DnaA antibody
[
[
[
[
[
[
[
DdnaA
+ -
+ -
+ -
+ -
+ -
+ -
+ -

## Slide 6
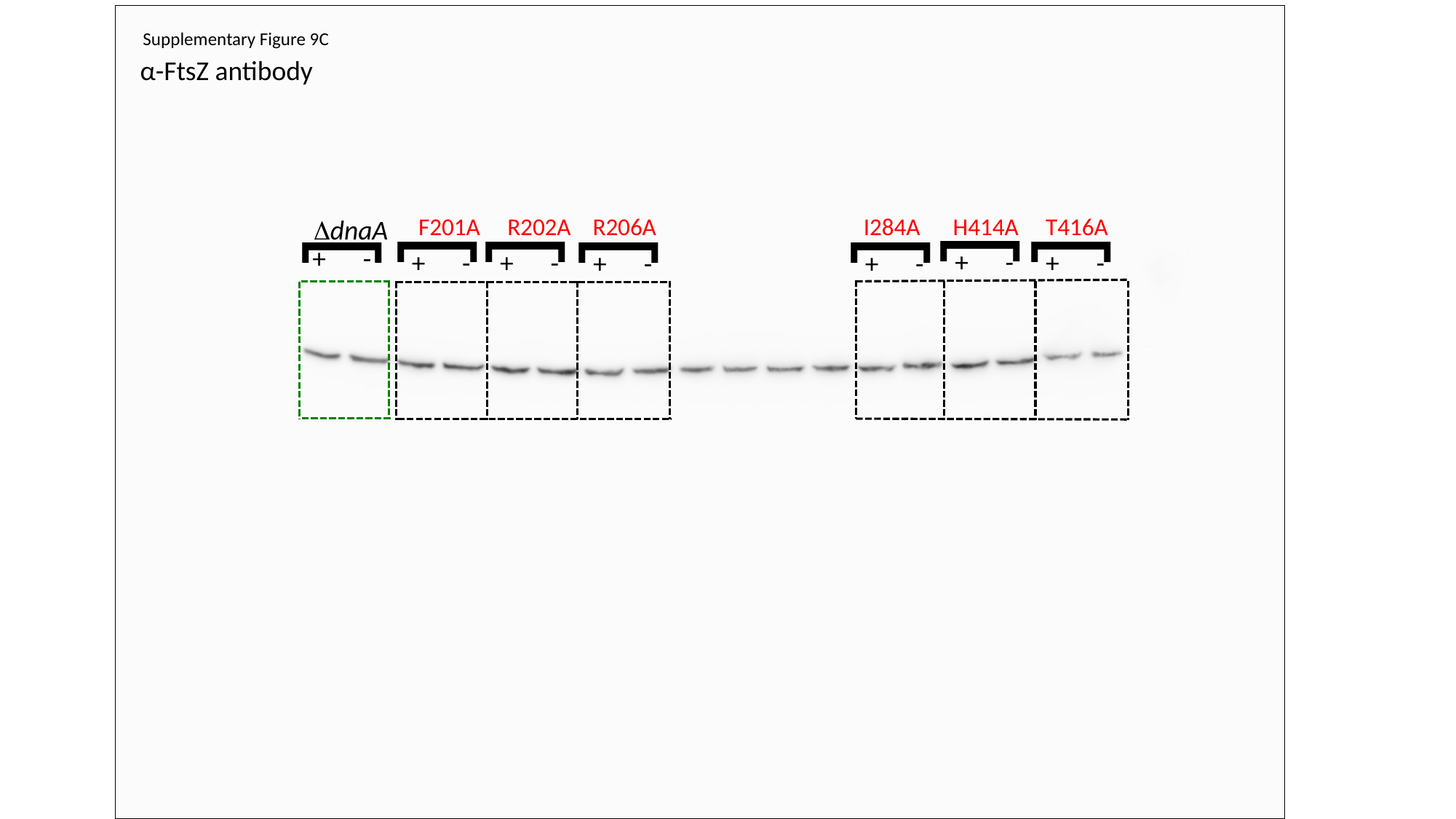

F201A R202A R206A I284A H414A T416A
Supplementary Figure 9C
α-FtsZ antibody
[
DdnaA
+ -
[
+ -
[
+ -
[
+ -
[
+ -
[
+ -
[
+ -

## Slide 7
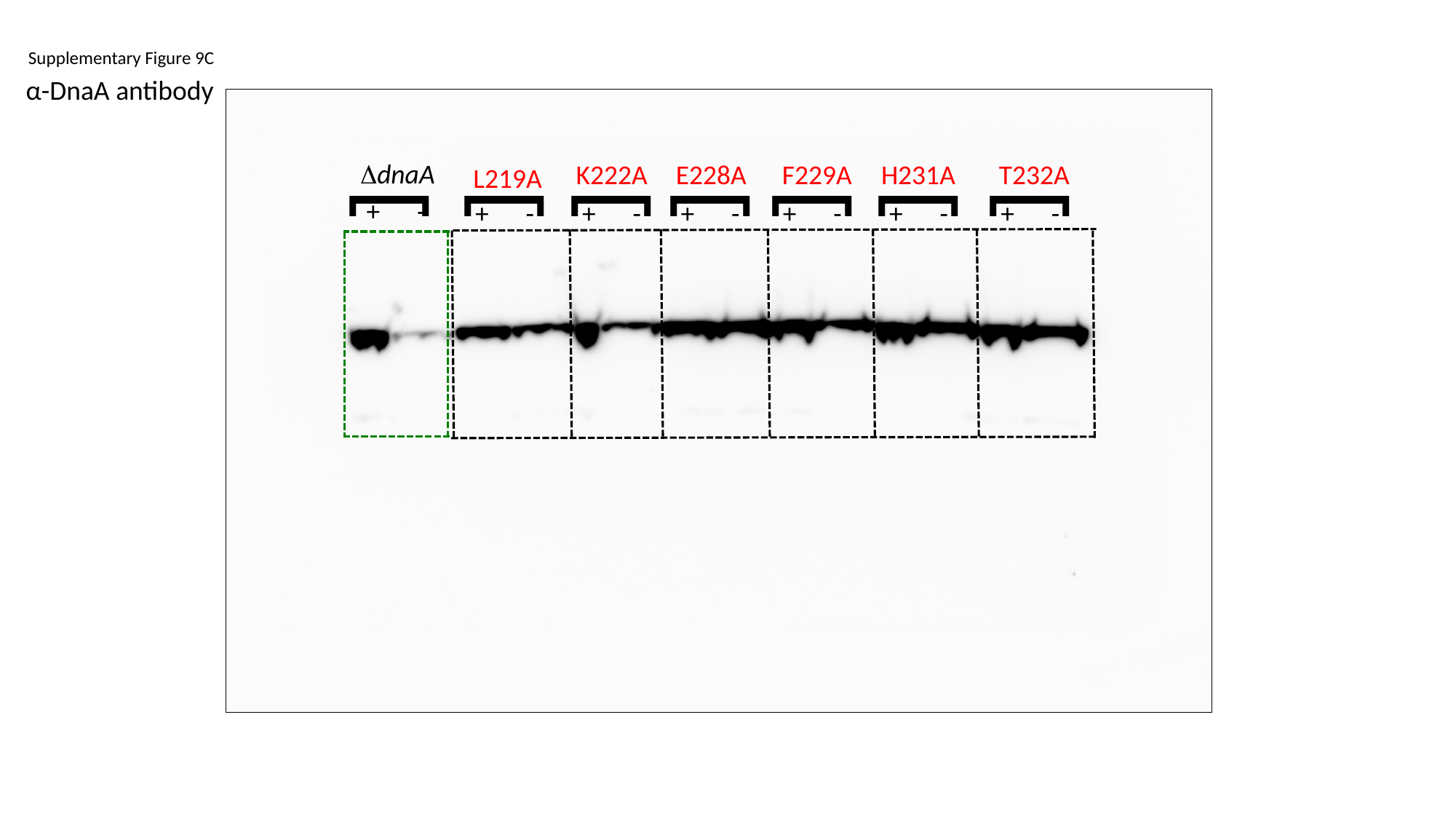

Supplementary Figure 9C
α-DnaA antibody
DdnaA
K222A
E228A
T232A
F229A
H231A
L219A
[
[
+ -
[
+ -
[
+ -
[
+ -
[
+ -
[
+ -
+ -
+ -
+ -
+ -
+ -
+ -
+ -

## Slide 8
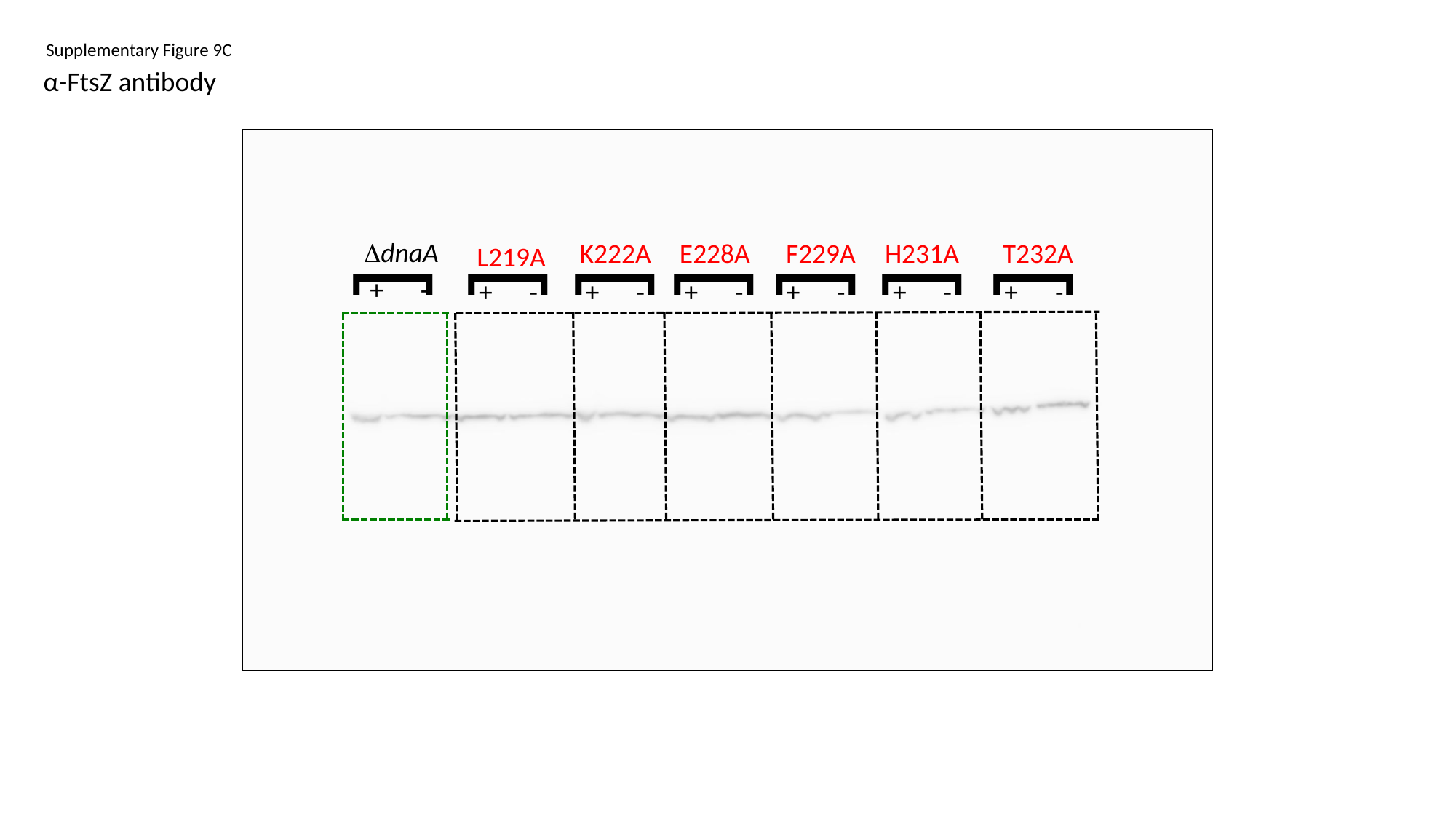

Supplementary Figure 9C
α-FtsZ antibody
DdnaA
K222A
E228A
T232A
F229A
H231A
L219A
[
[
+ -
[
+ -
[
+ -
[
+ -
[
+ -
[
+ -
+ -

## Slide 9
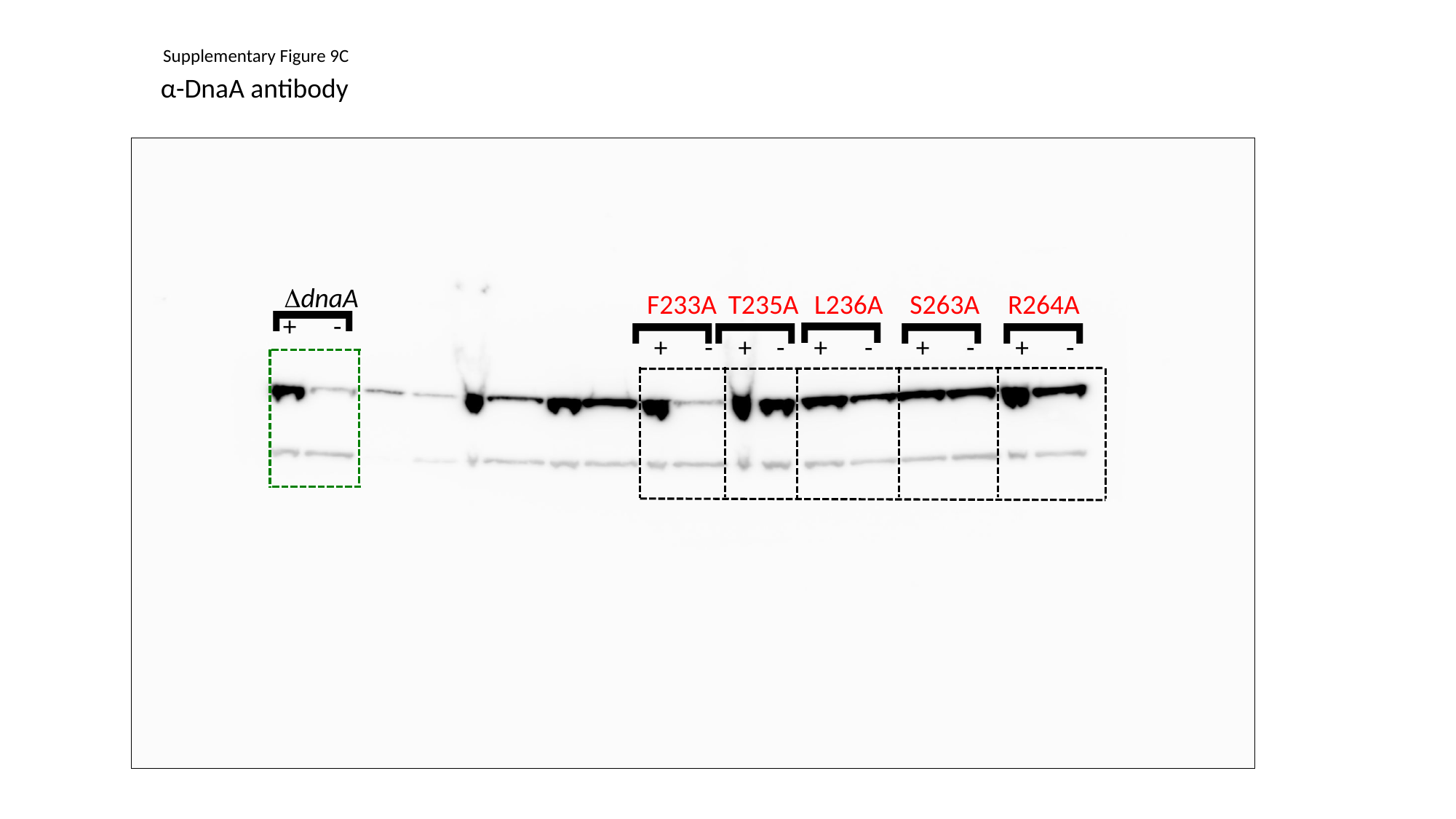

Supplementary Figure 9C
α-DnaA antibody
[
[
DdnaA
+ -
F233A
R264A
T235A
L236A
S263A
[
[
[
[
+ -
+ -
+ -
+ -
+ -

## Slide 10
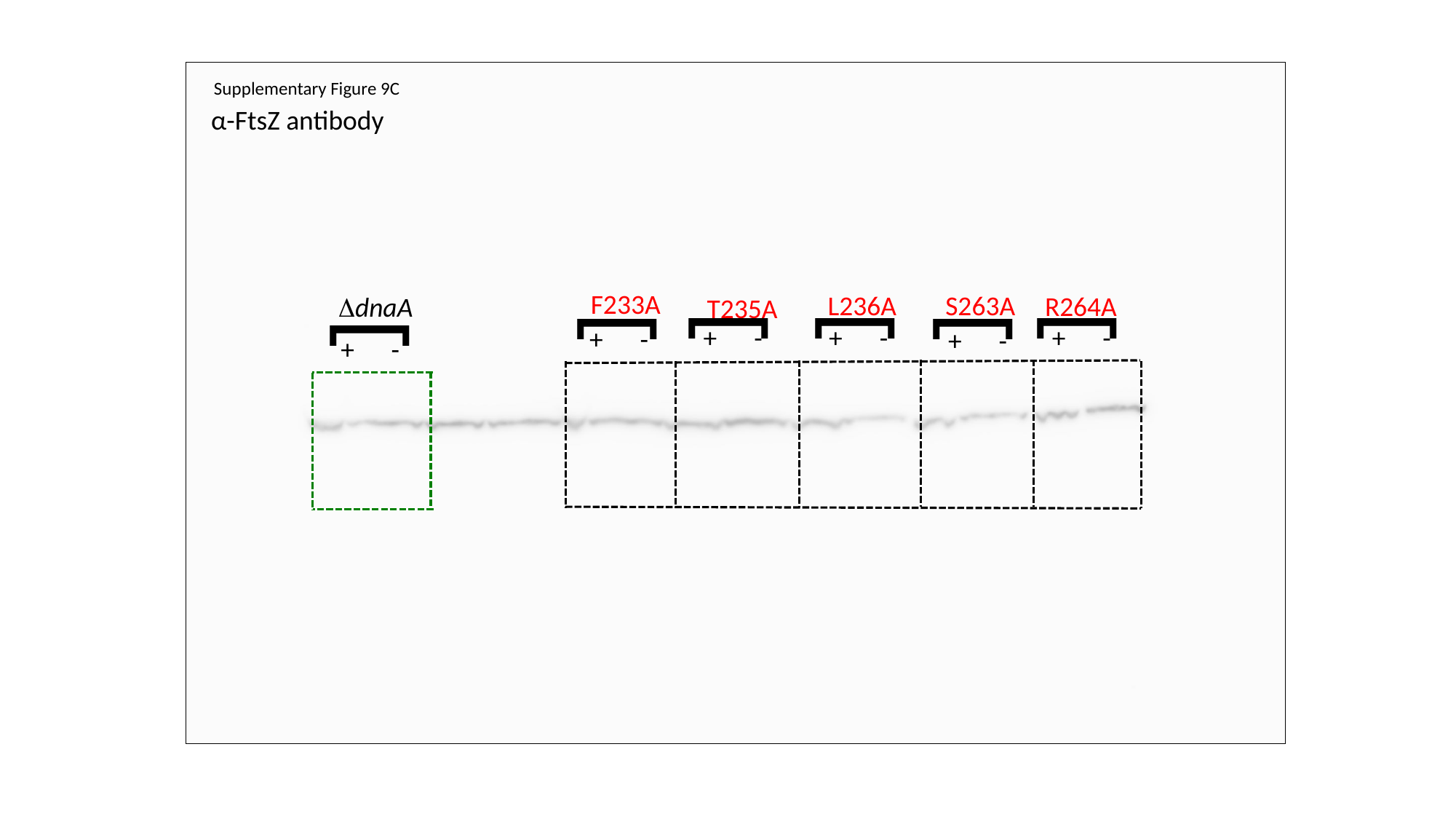

Supplementary Figure 9C
α-FtsZ antibody
[
F233A
L236A
S263A
R264A
T235A
DdnaA
[
[
[
[
[
+ -
+ -
+ -
+ -
+ -
+ -
&FtsZ

## Slide 11
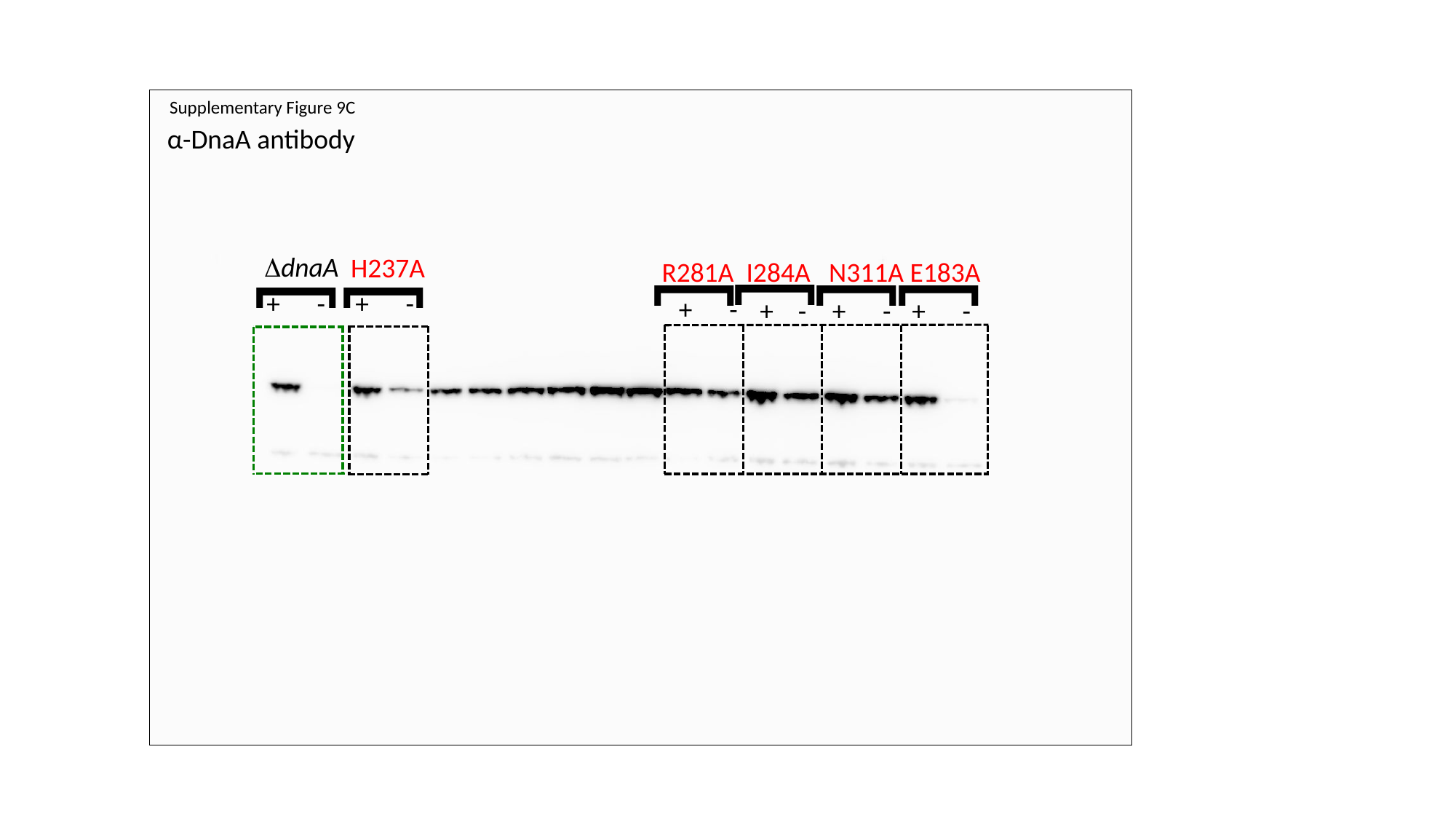

Supplementary Figure 9C
α-DnaA antibody
[
[
[
DdnaA
H237A
R281A I284A N311A E183A
[
[
[
+ -
+ -
+ -
+ -
+ -
+ -

## Slide 12
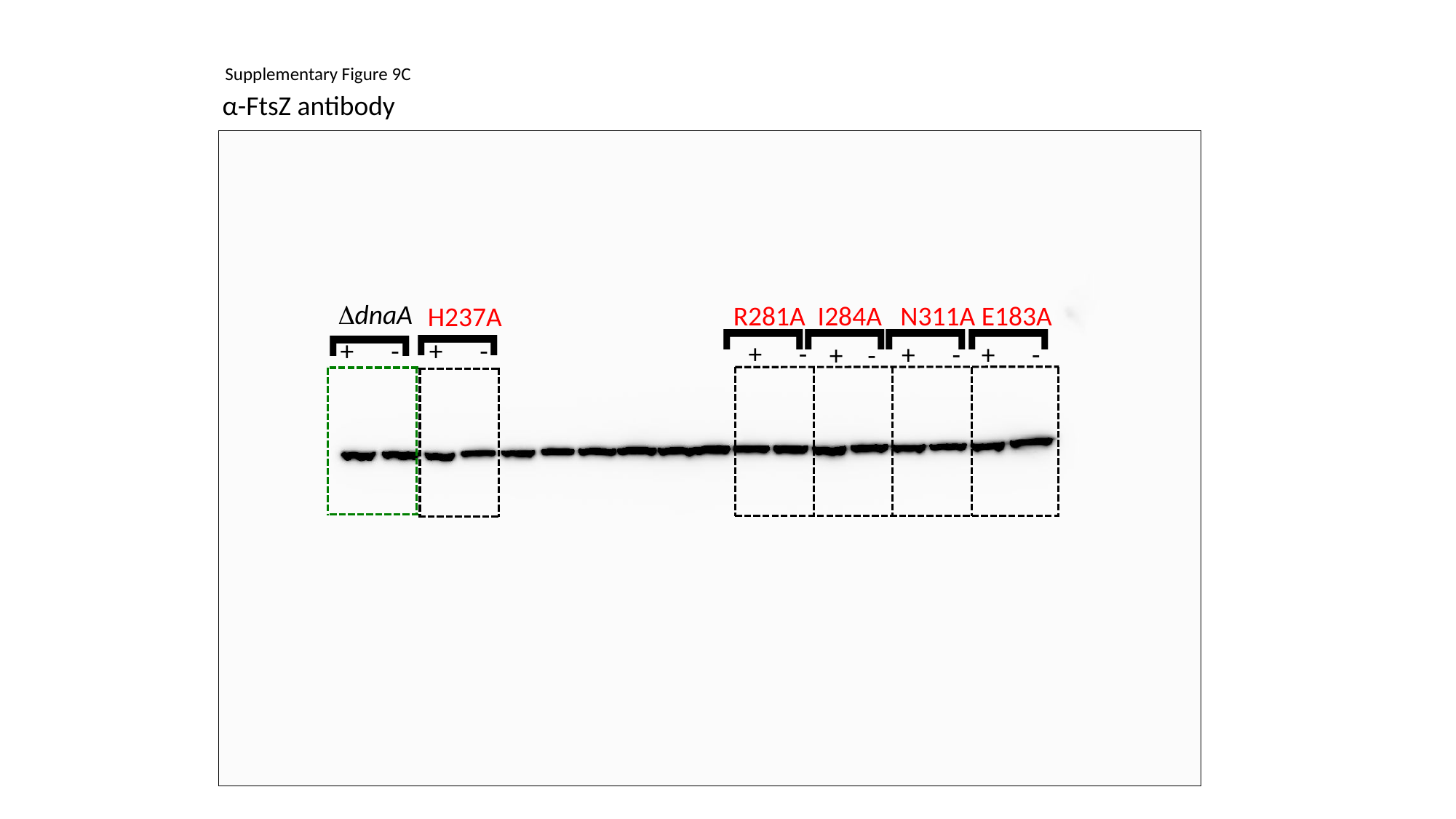

Supplementary Figure 9C
α-FtsZ antibody
[
[
[
DdnaA
R281A I284A N311A E183A
H237A
[
[
[
+ -
+ -
+ -
+ -
+ -
+ -

## Slide 13
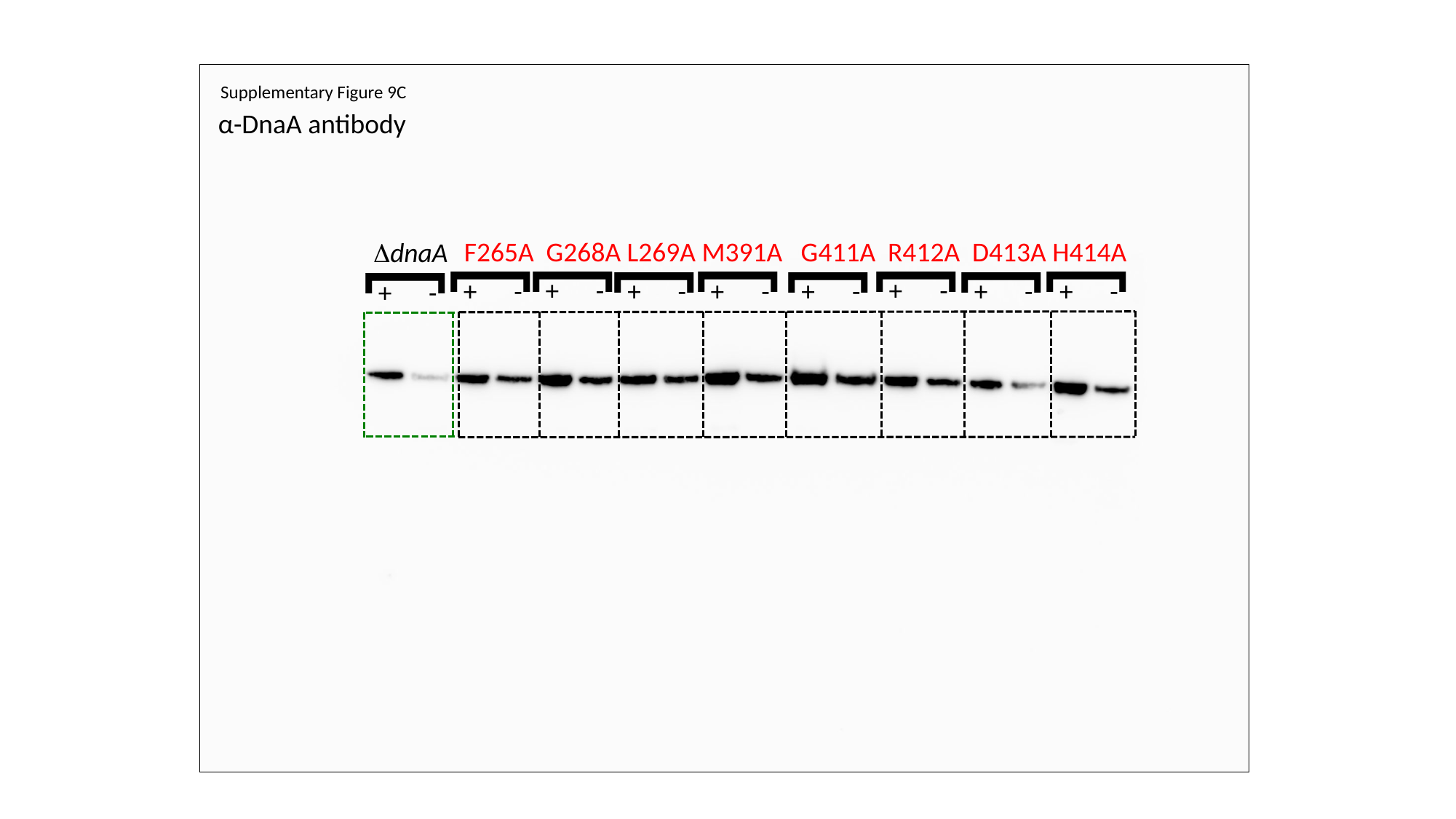

Supplementary Figure 9C
α-DnaA antibody
[
F265A G268A L269A M391A G411A R412A D413A H414A
DdnaA
[
+ -
[
+ -
[
+ -
[
+ -
[
+ -
[
+ -
[
+ -
[
+ -
+ -

## Slide 14
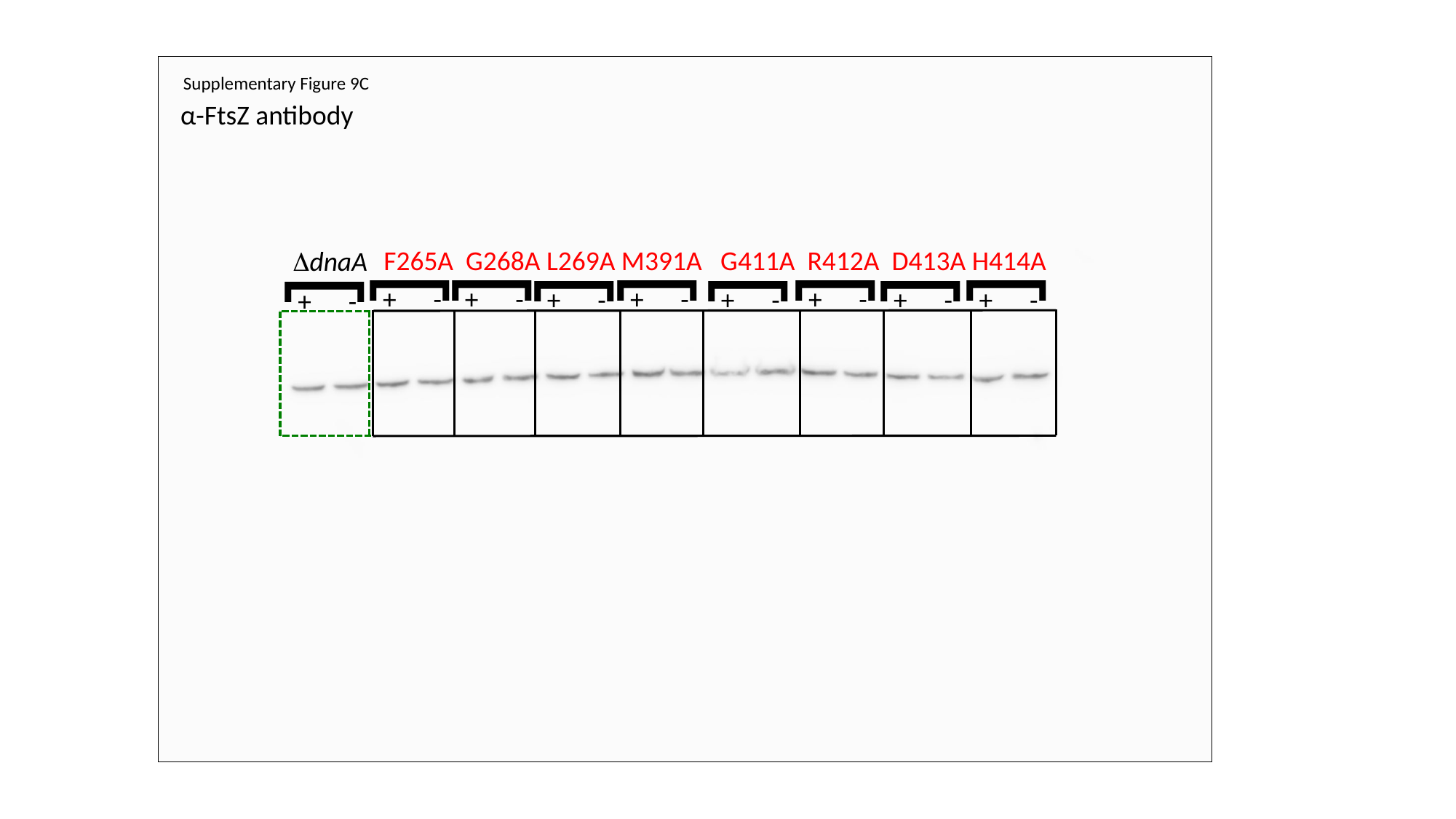

Supplementary Figure 9C
α-FtsZ antibody
[
F265A G268A L269A M391A G411A R412A D413A H414A
DdnaA
[
+ -
[
+ -
[
+ -
[
+ -
[
+ -
[
+ -
[
+ -
[
+ -
+ -

## Slide 15
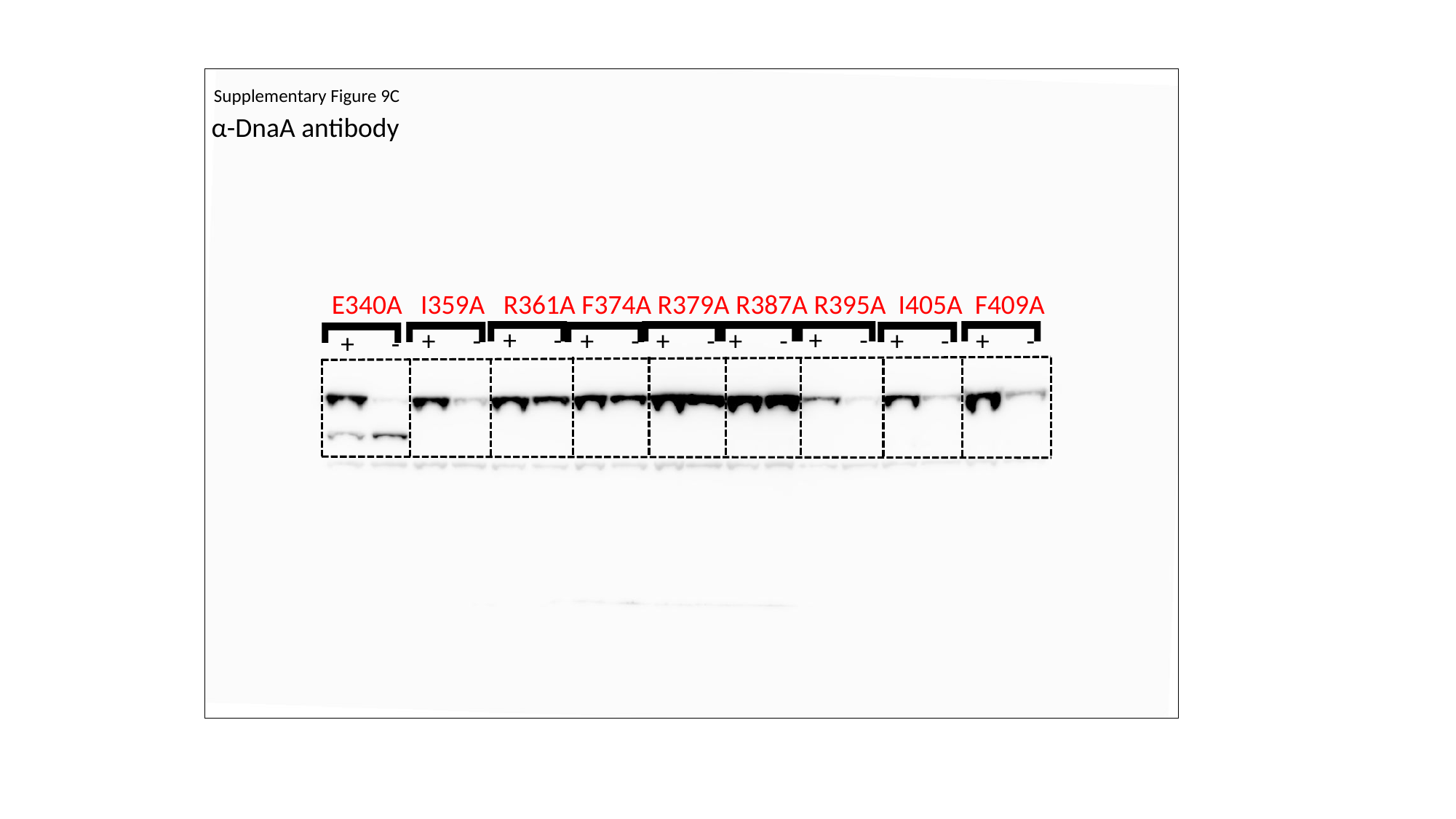

Supplementary Figure 9C
α-DnaA antibody
[
E340A I359A R361A F374A R379A R387A R395A I405A F409A
[
+ -
[
+ -
[
+ -
[
+ -
[
+ -
[
+ -
[
+ -
[
+ -
+ -

## Slide 16
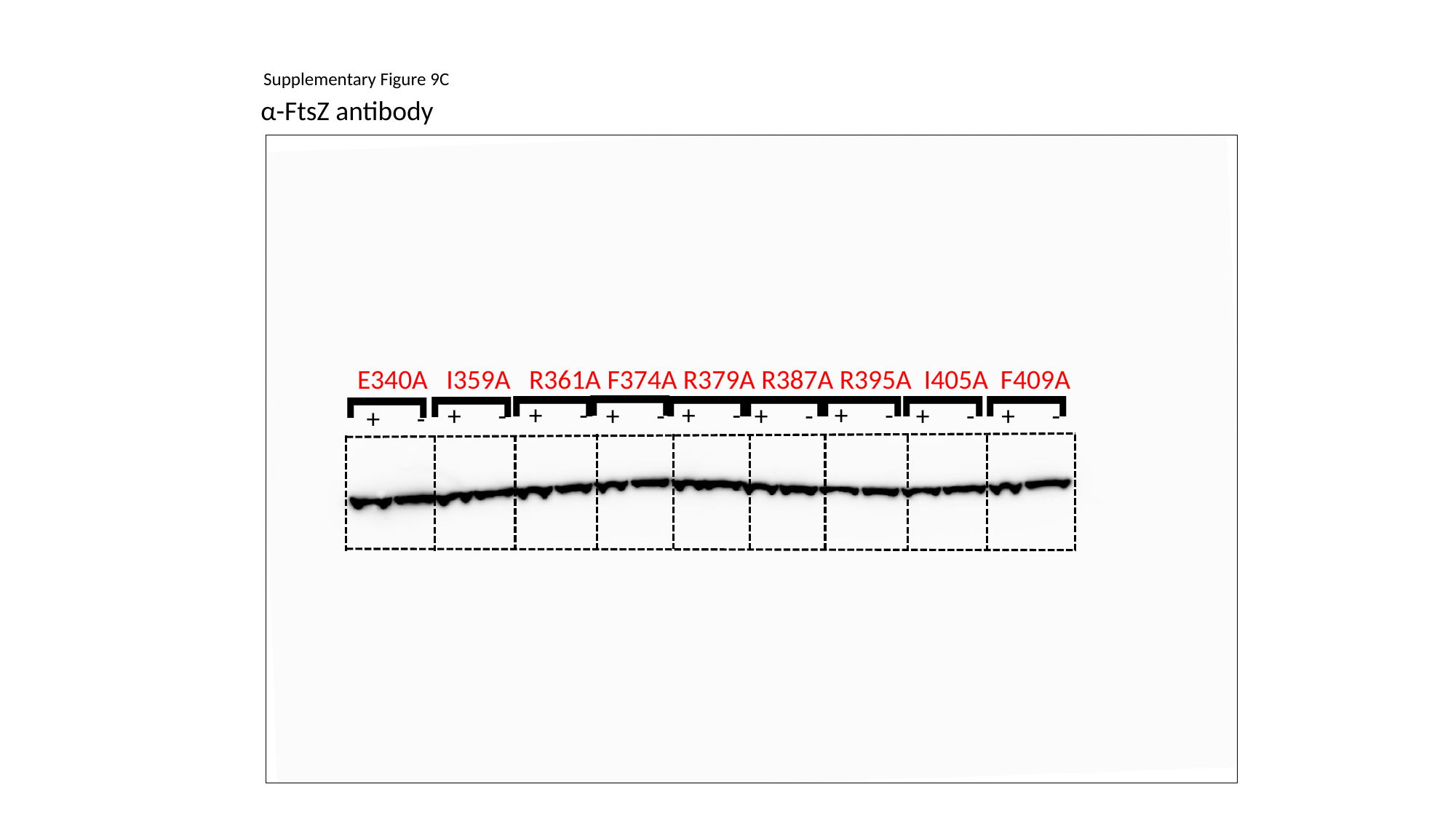

Supplementary Figure 9C
α-FtsZ antibody
[
E340A I359A R361A F374A R379A R387A R395A I405A F409A
[
+ -
[
+ -
[
+ -
[
+ -
[
+ -
[
+ -
[
+ -
[
+ -
+ -

## Slide 17
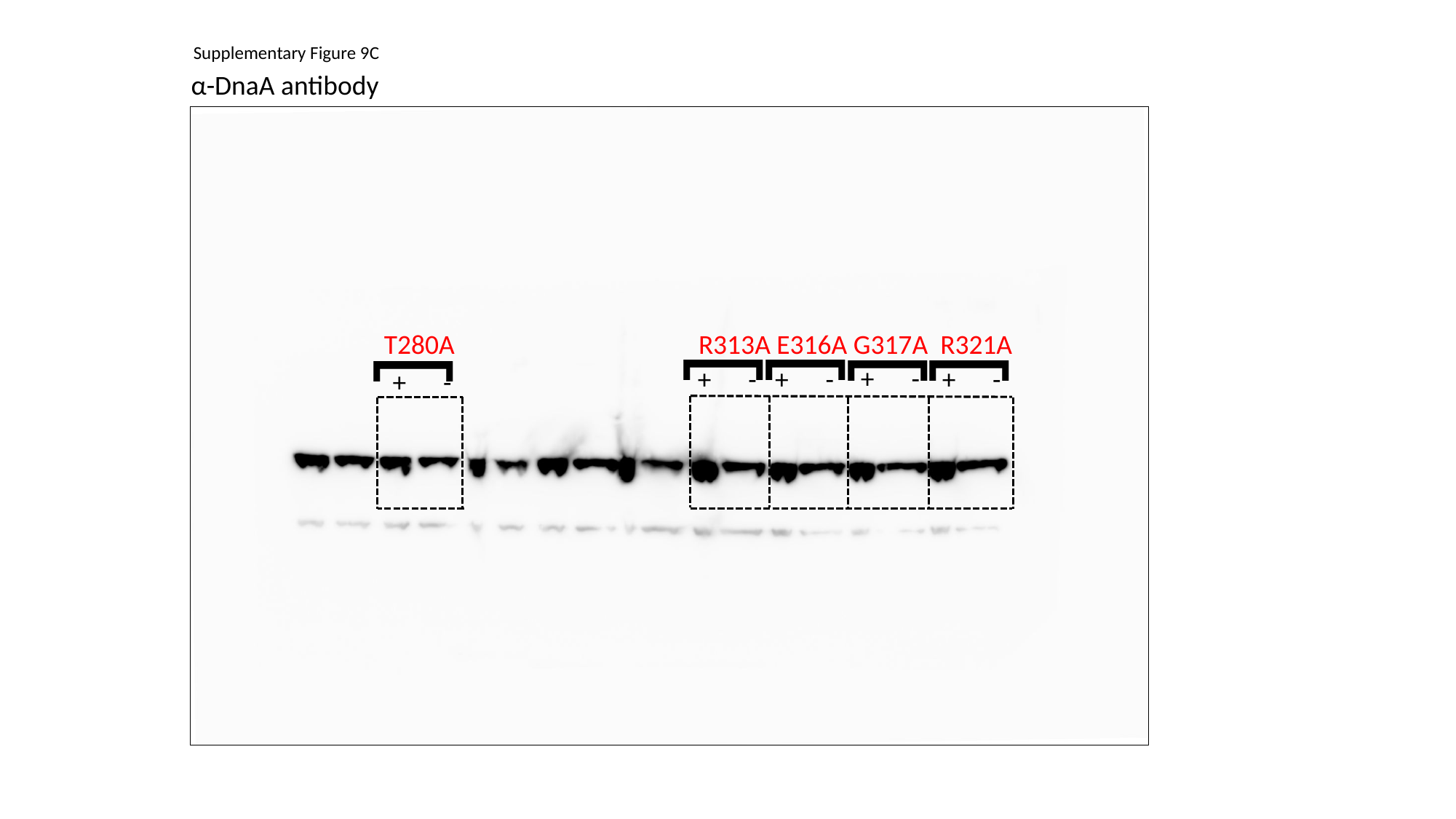

Supplementary Figure 9C
α-DnaA antibody
[
T280A
R313A E316A G317A R321A
[
+ -
[
+ -
[
+ -
[
+ -
+ -

## Slide 18
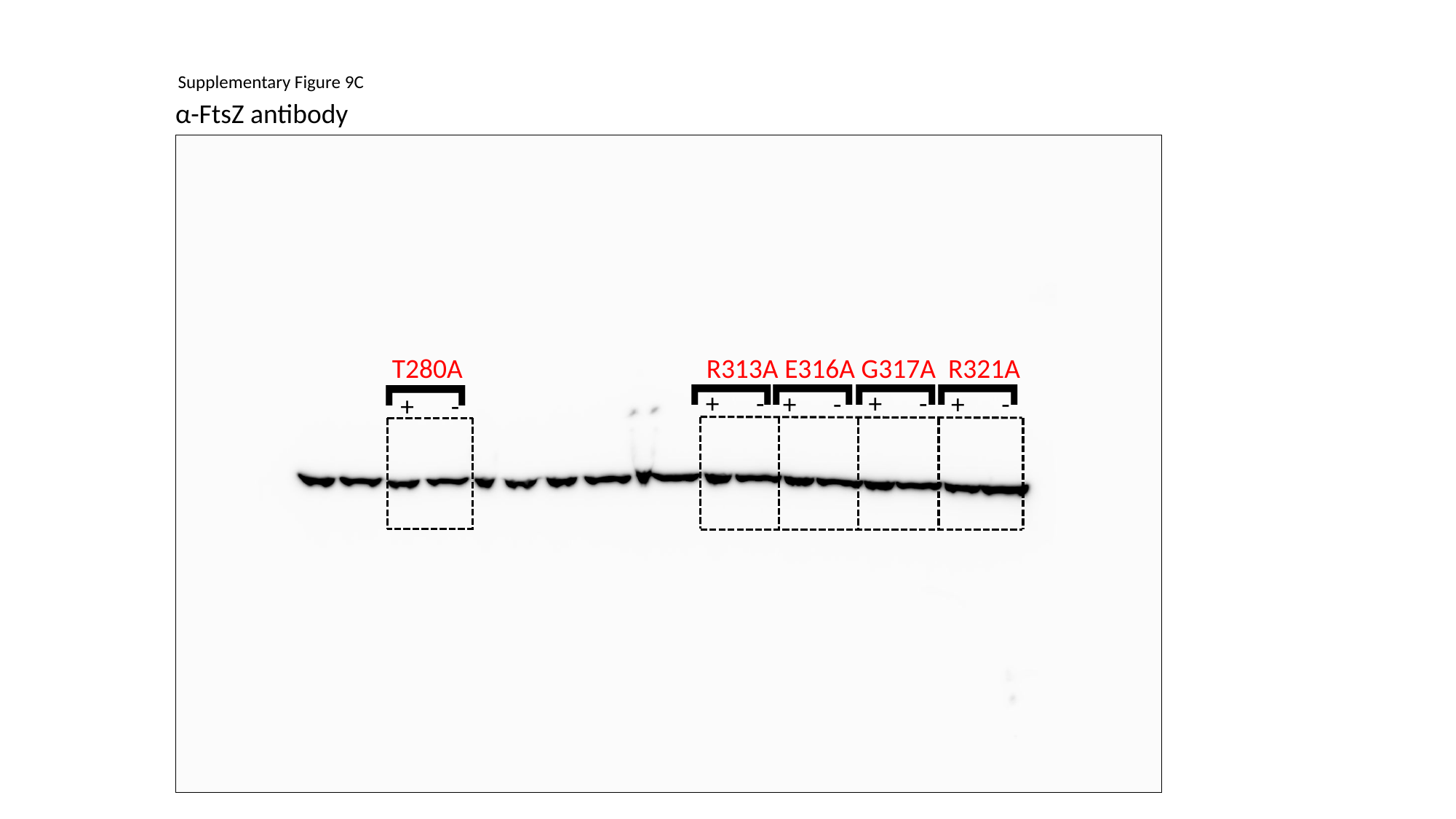

Supplementary Figure 9C
α-FtsZ antibody
[
T280A
R313A E316A G317A R321A
[
+ -
[
+ -
[
+ -
[
+ -
+ -

## Slide 19
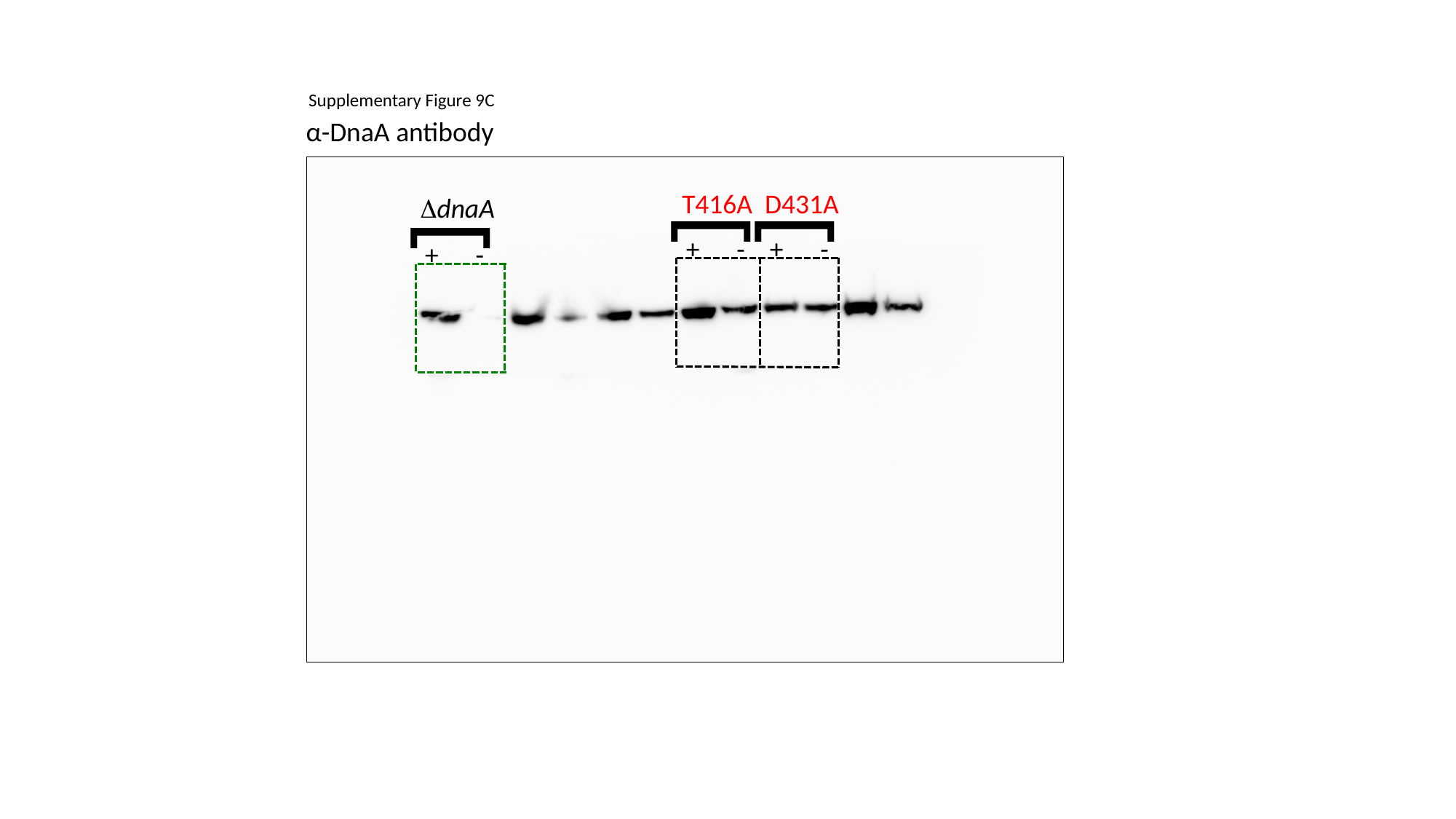

Supplementary Figure 9C
α-DnaA antibody
T416A D431A
DdnaA
[
+ -
[
+ -
[
+ -

## Slide 20
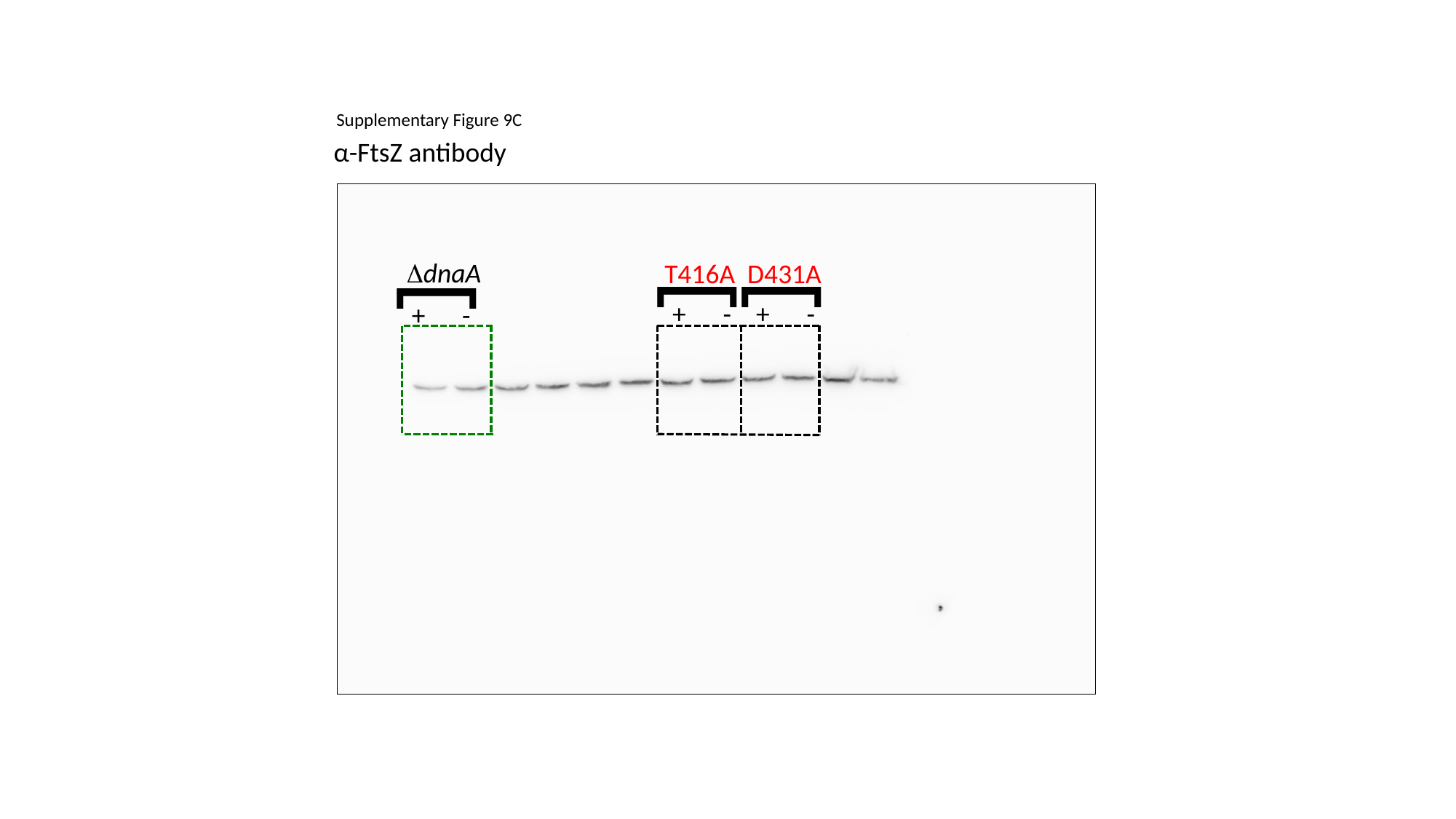

Supplementary Figure 9C
α-FtsZ antibody
DdnaA
T416A D431A
[
+ -
[
+ -
[
+ -
