# Supplementary material for: The bacterial replication origin BUS promotes nucleobase capture
Source: Nat Commun. 2023 Dec 14;14:8339. doi: 10.1038/s41467-023-43823-w (PMC10721633; doi:10.1038/s41467-023-43823-w)

Supplementary Figure 5C

Coomassie stained SDS-PAGE

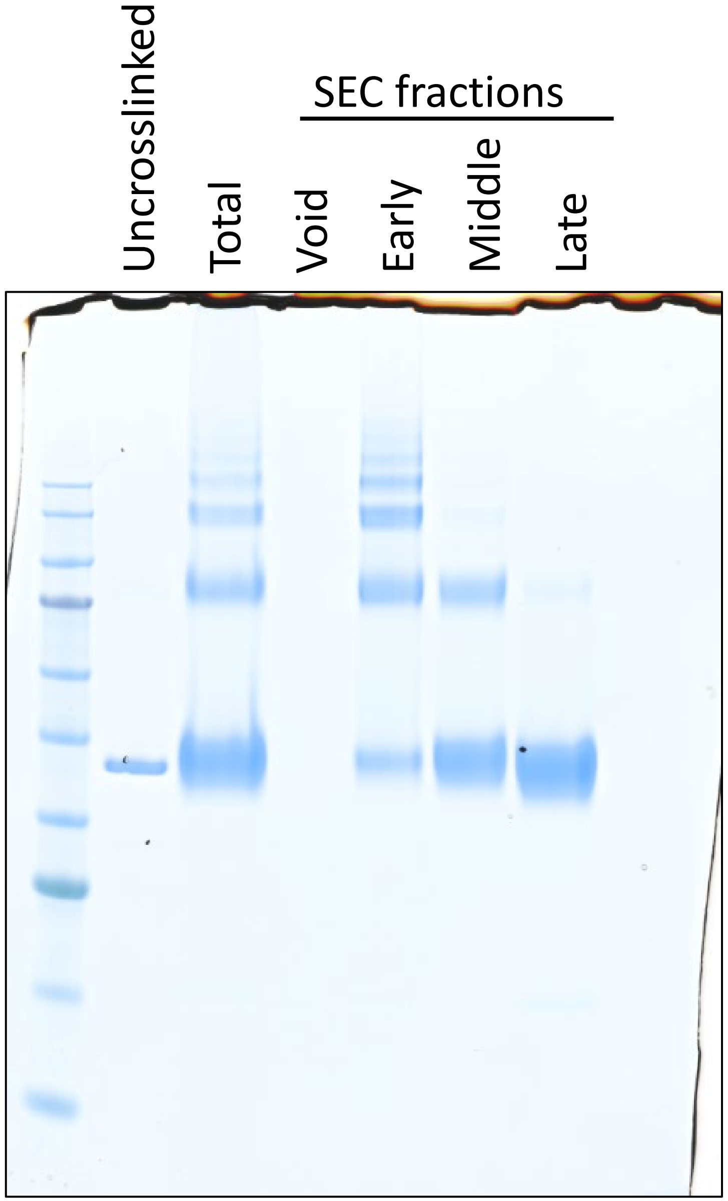

# Confirmation of polyclonal antibody specificity (related to Supplementary Figure 9C)

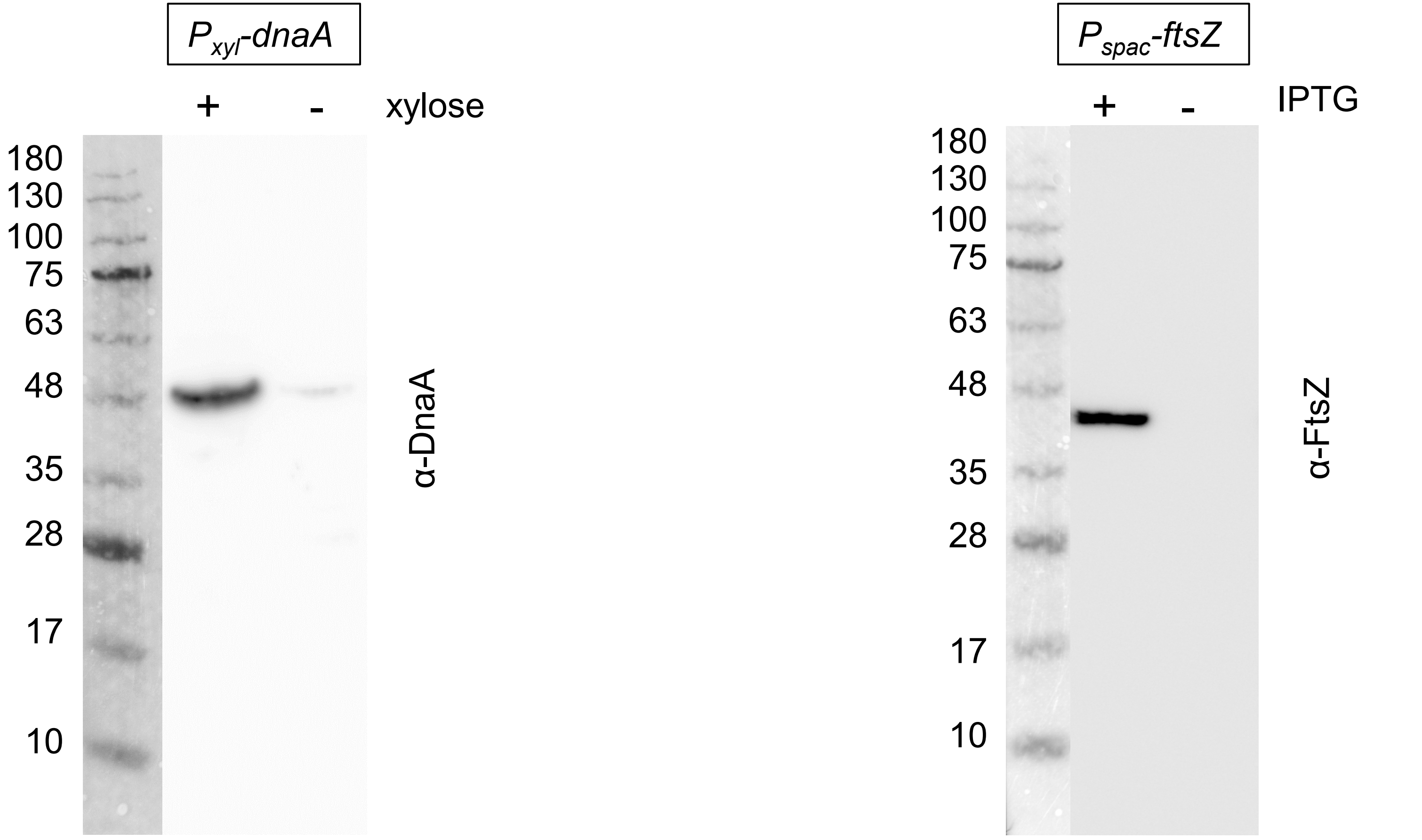


To demonstrate the specificity of the polyclonal DnaA antibody used in this study, strain FDS257 was grown at 37°C in LB supplemented with or without 1% xylose until $A_{600}$ = 1.0. Cells were harvested, total proteins extracted, and the samples ran on SDS-PAGE. Separated proteins were transferred to PVDF membrane and probed with using antibodies against DnaA.

To demonstrate the specificity of the polyclonal FtsZ antibody used in this study, strain HM110 (*trpC2 $P_{spac}$-ftsZ)* was grown at 37°C in LB supplemented with or without 1 mM IPTG until $A_{600}$ = 1.0. Cells were harvested, total proteins extracted, and the samples ran on SDS-PAGE. Separated proteins were transferred to PVDF membrane and probed with using antibodies against FtsZ.

Protein molecular weight markers (kDa) are shown on the left (Abcamab #116027). DnaA has a molecular weight of 50.7 kDa. FtsZ has a molecular weight of 40.2 kDa.

Supplementary Figure 9C

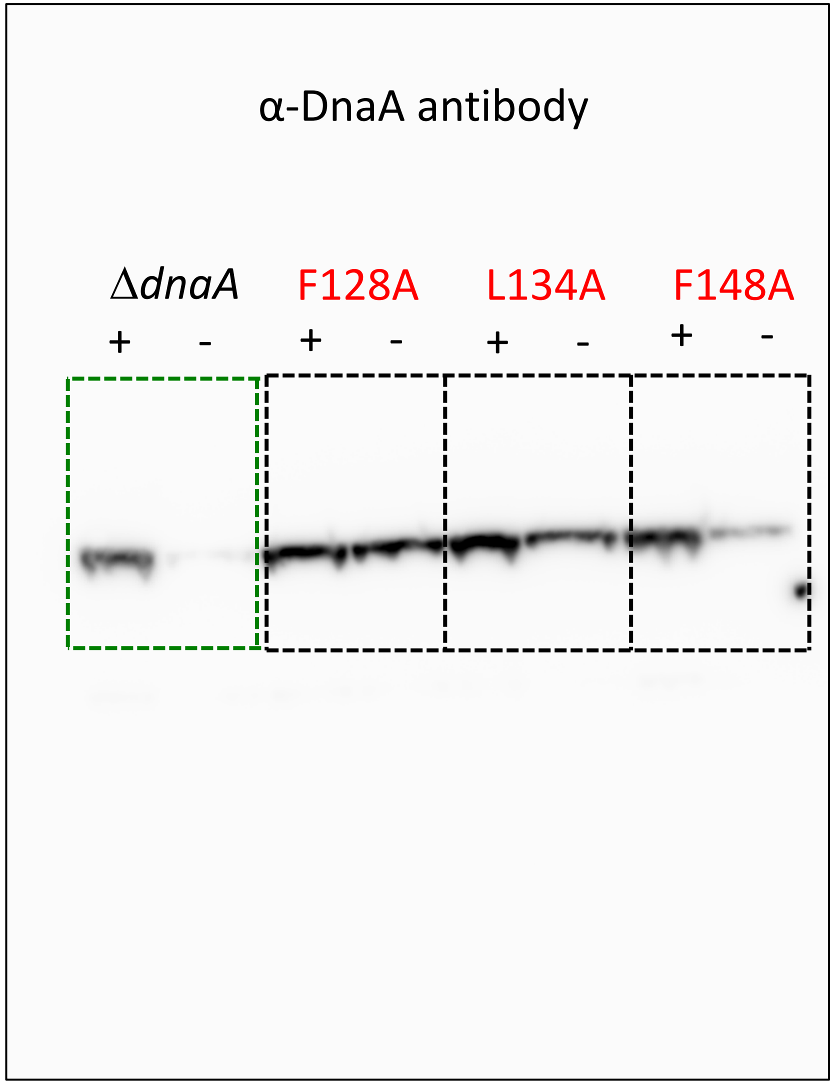


Supplementary Figure 9C

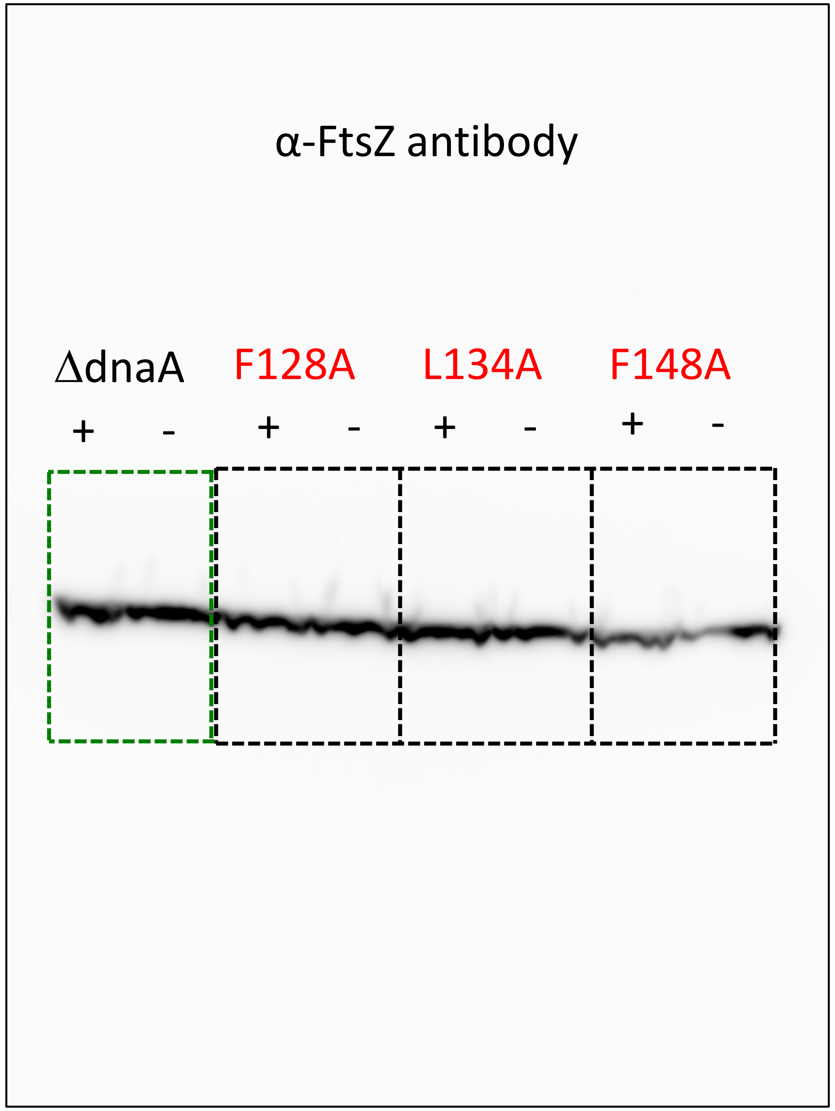

Supplementary Figure 9C

α-DnaA antibody

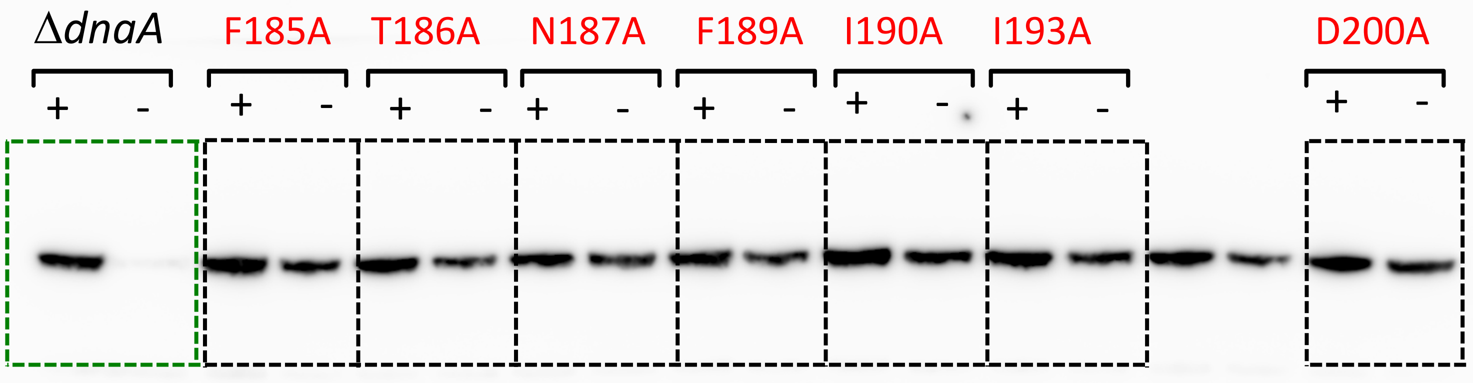

Supplementary Figure 9C

α-FtsZ antibody

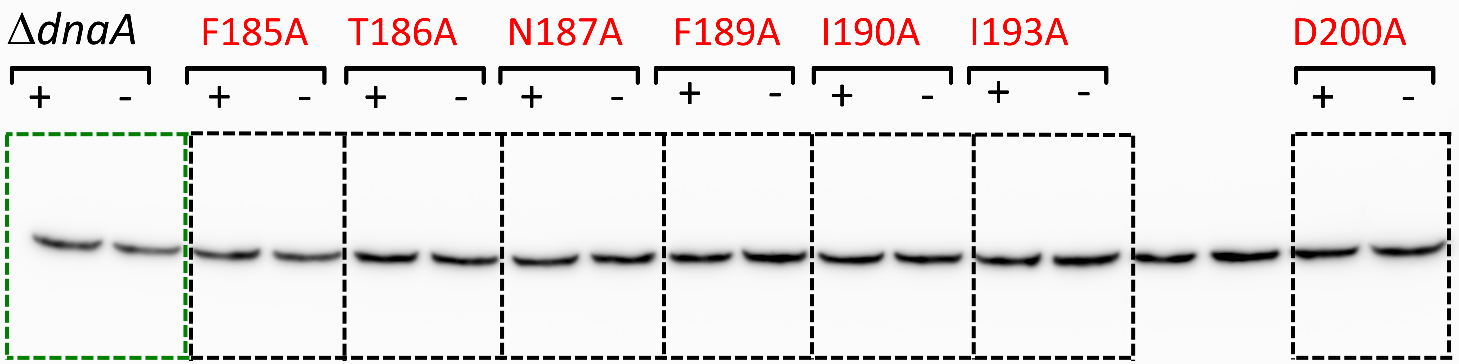

Supplementary Figure 9C

α-DnaA antibody

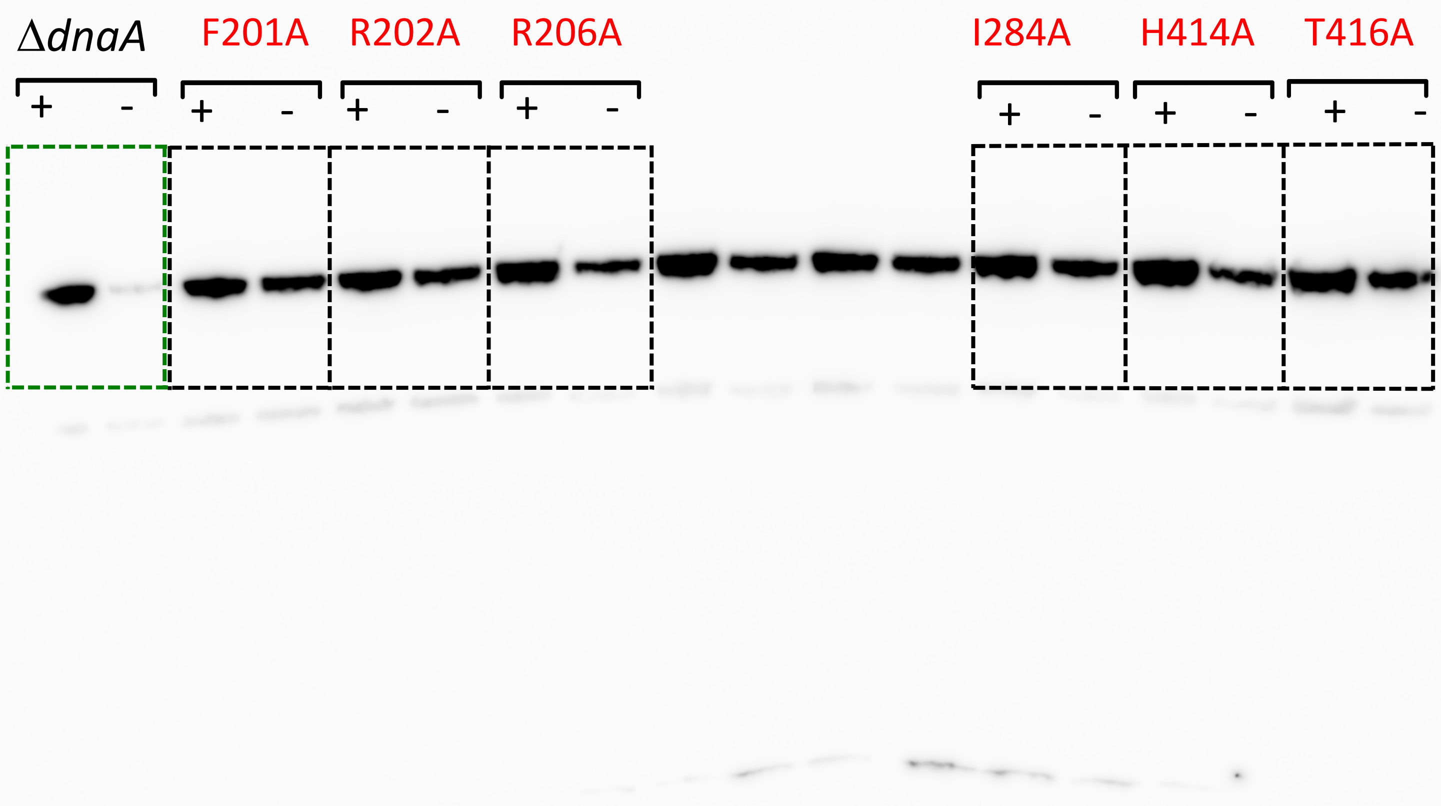

Supplementary Figure 9C

α-FtsZ antibody

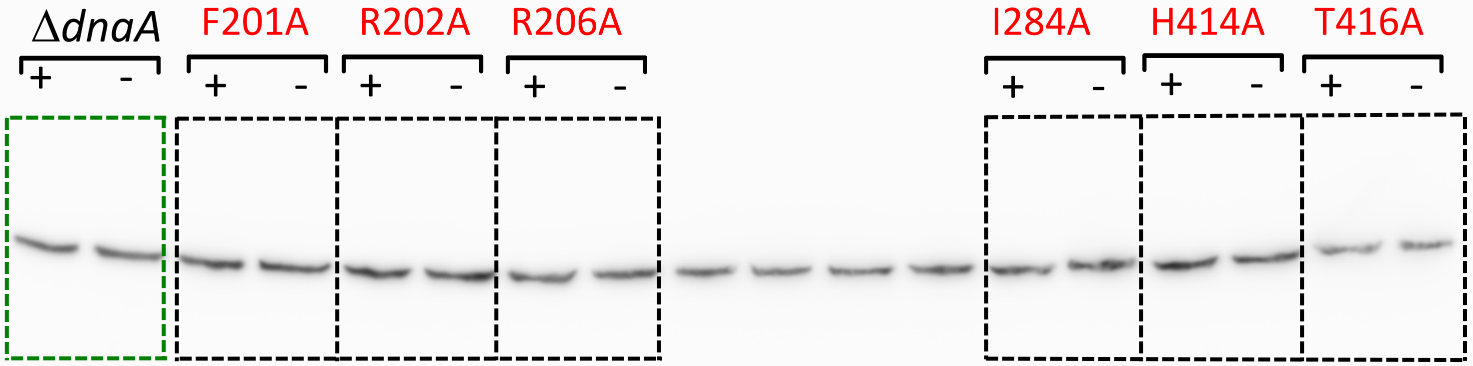

Supplementary Figure 9C

α-DnaA antibody

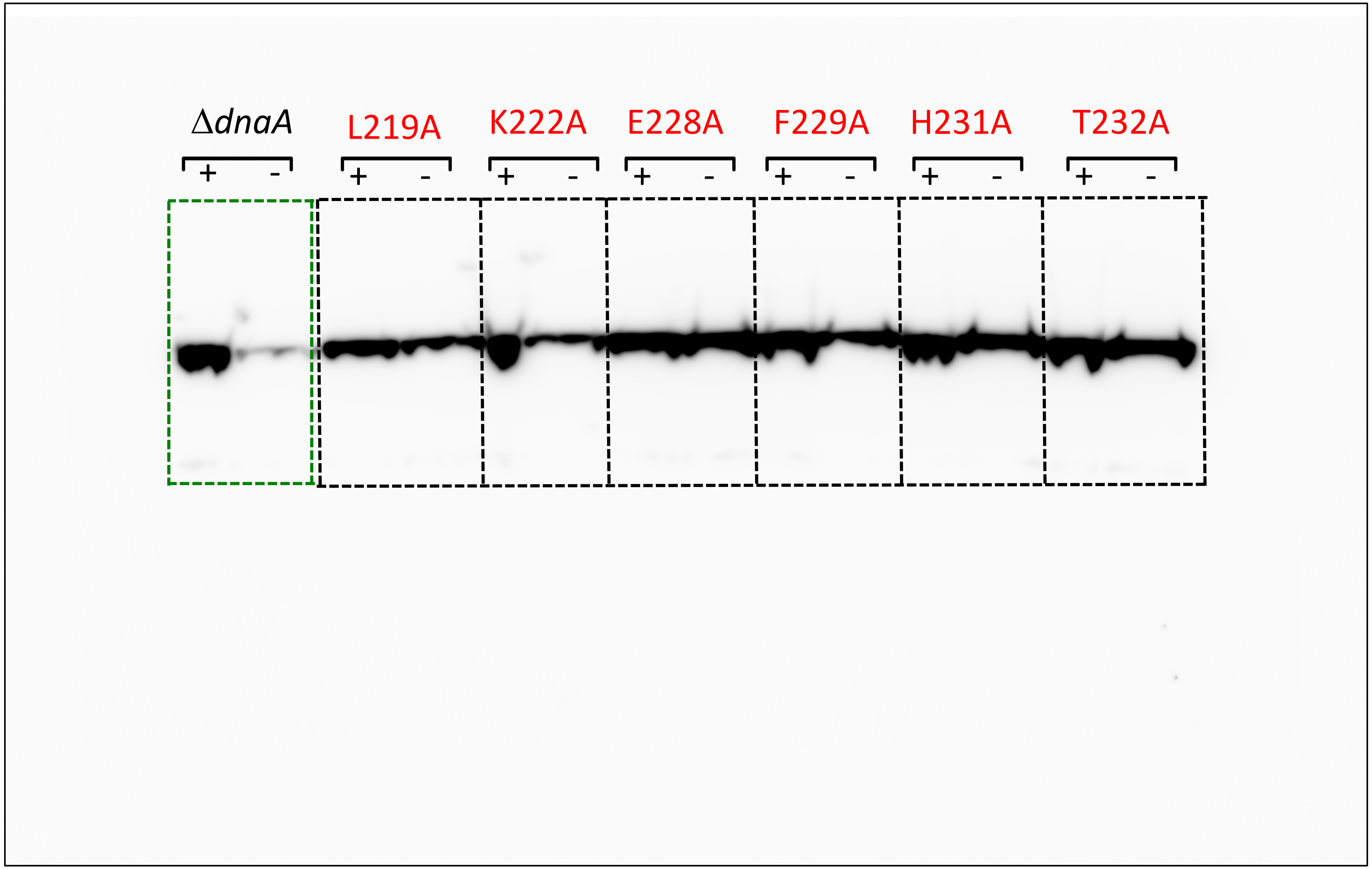

Supplementary Figure 9C

α-FtsZ antibody

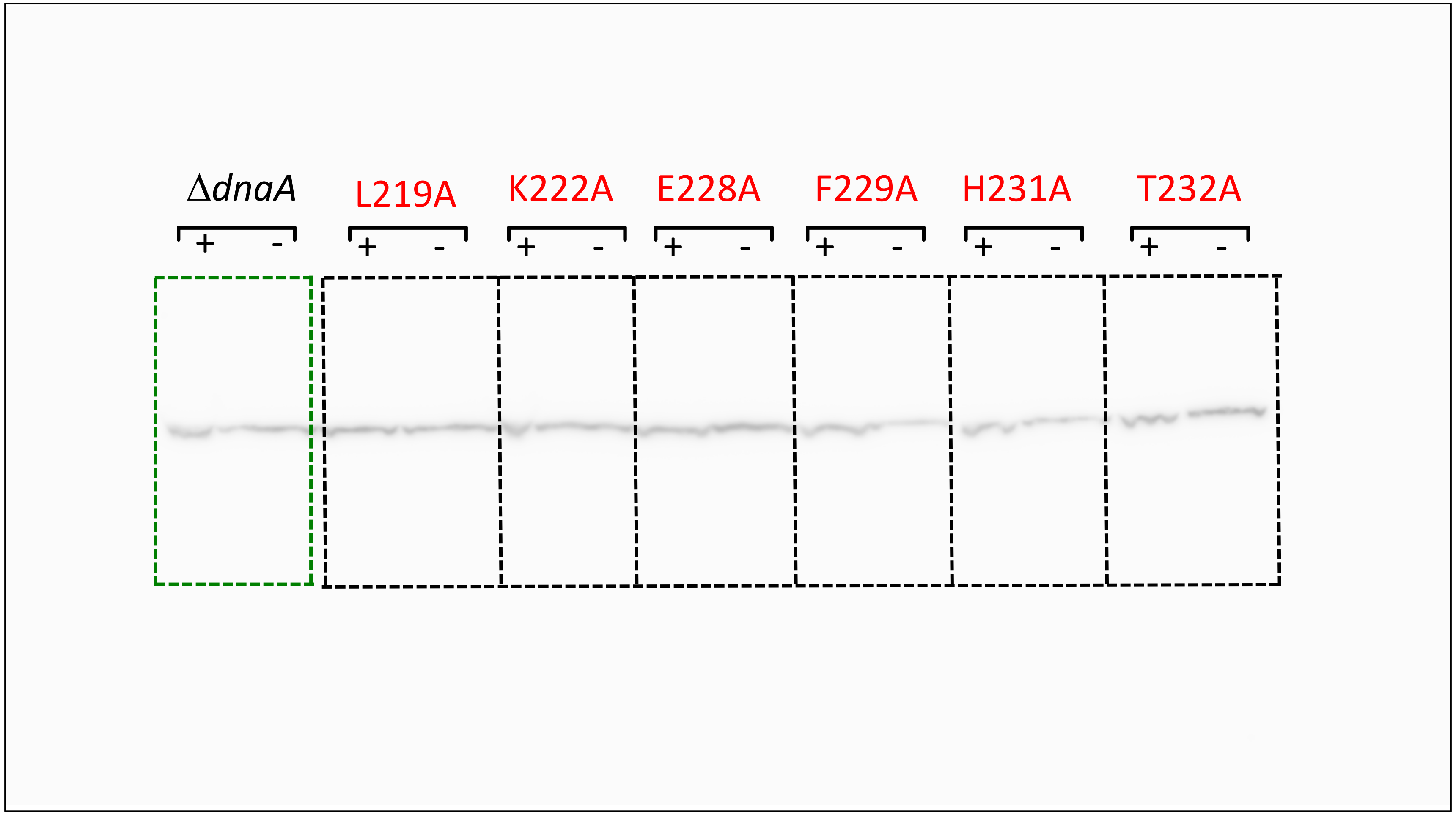

Supplementary Figure 9C

α-DnaA antibody

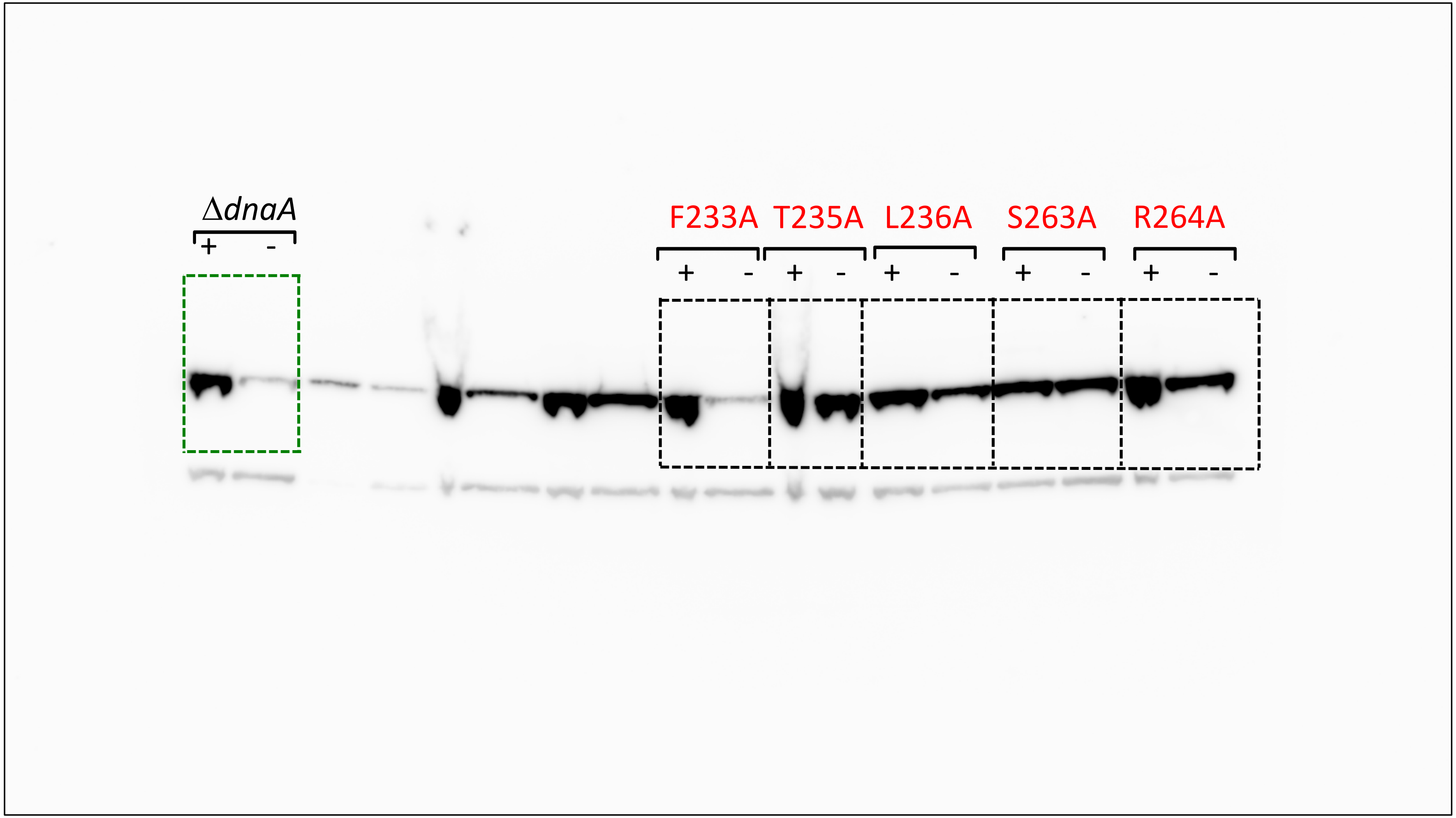

Supplementary Figure 9C

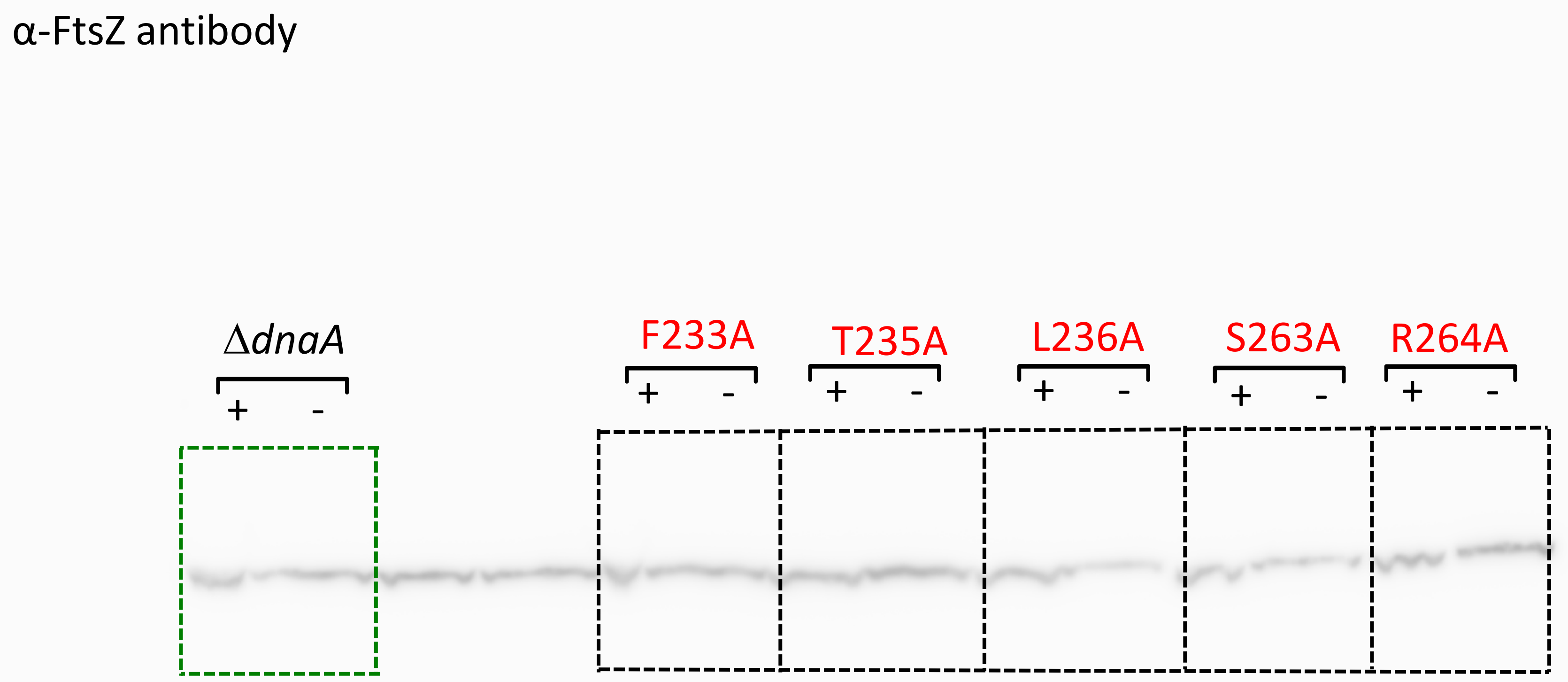

Supplementary Figure 9C

α-DnaA antibody

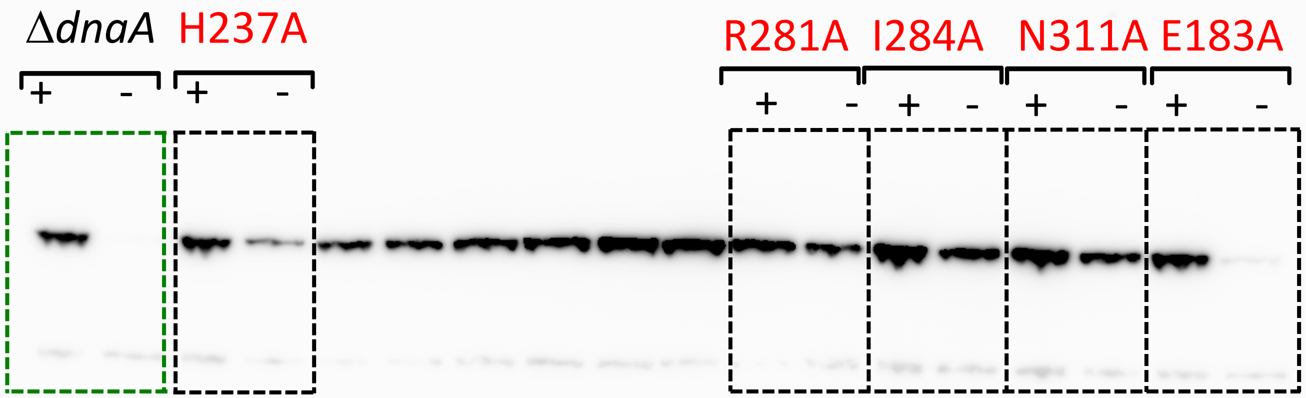

Supplementary Figure 9C

α-FtsZ antibody

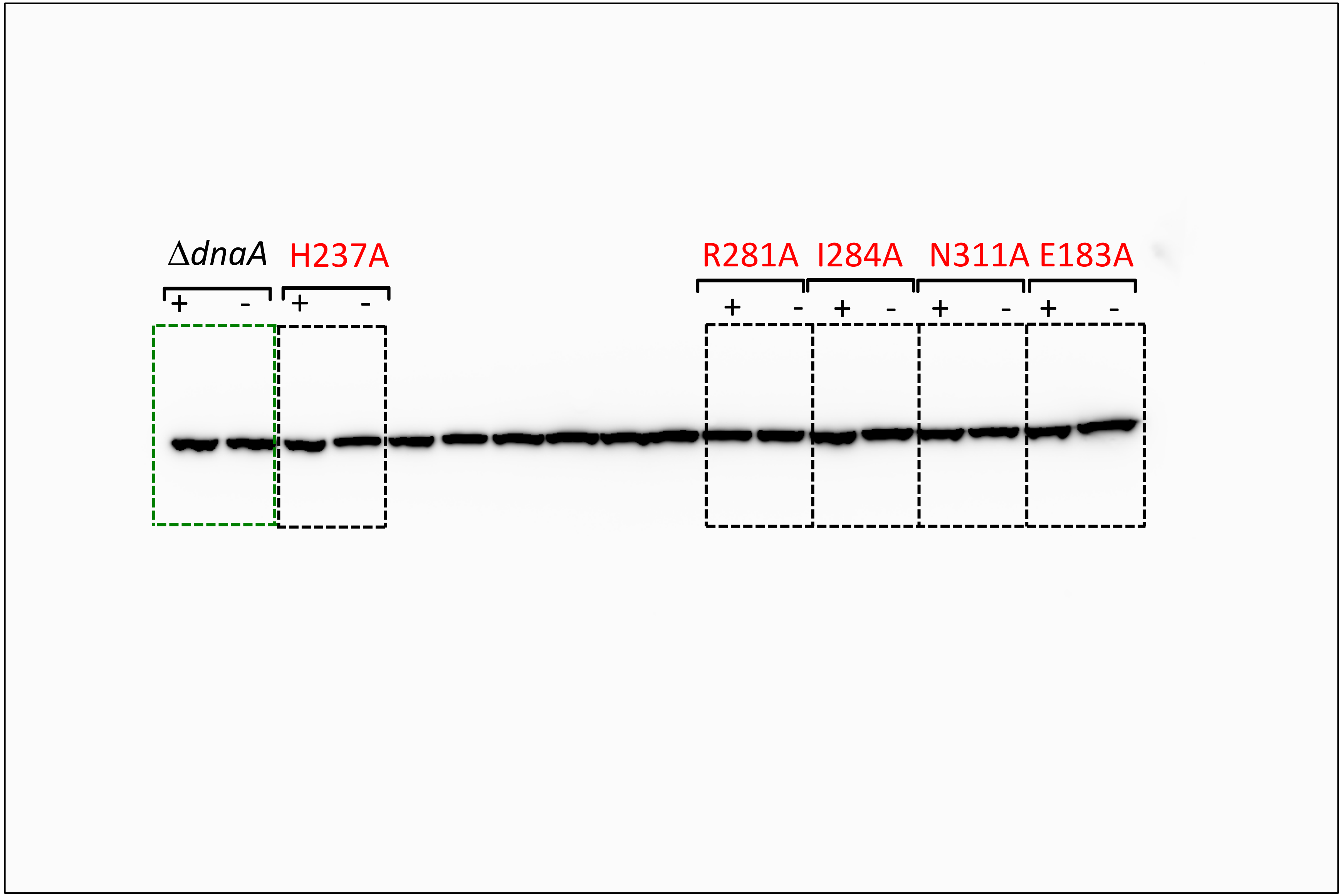

Supplementary Figure 9C

α-DnaA antibody

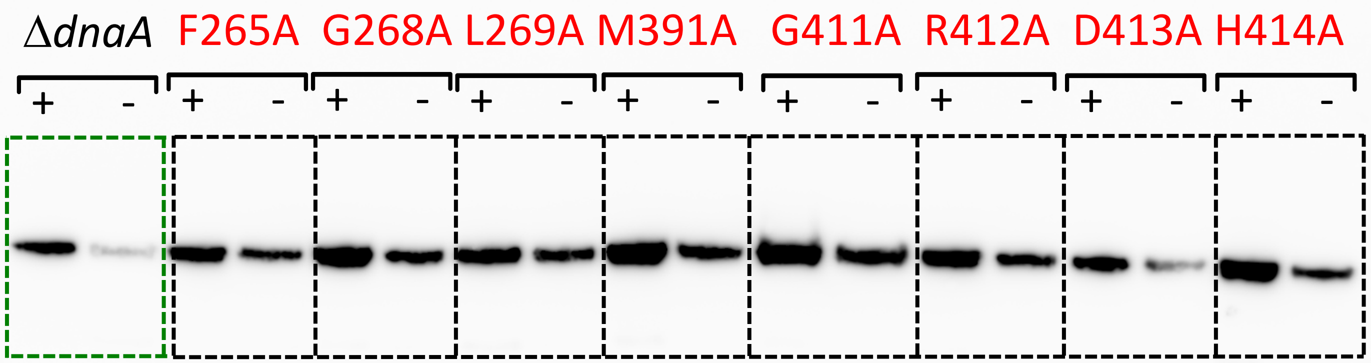

Supplementary Figure 9C

α-FtsZ antibody

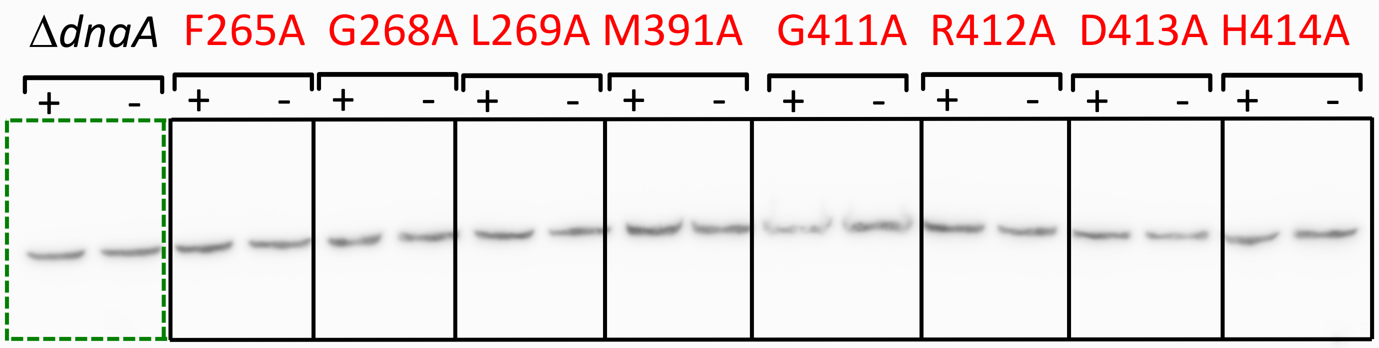

Supplementary Figure 9C

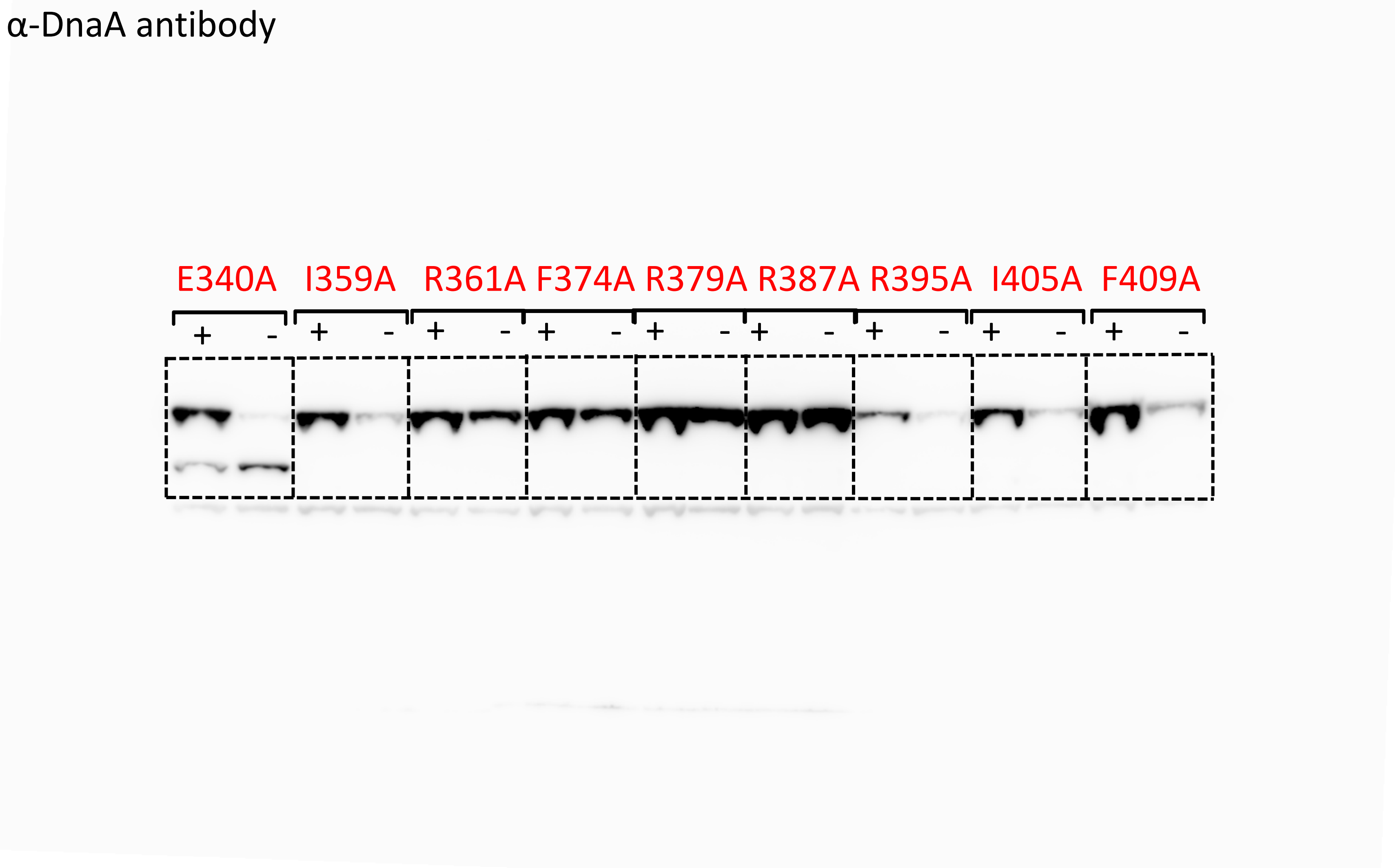

Supplementary Figure 9C

α-FtsZ antibody

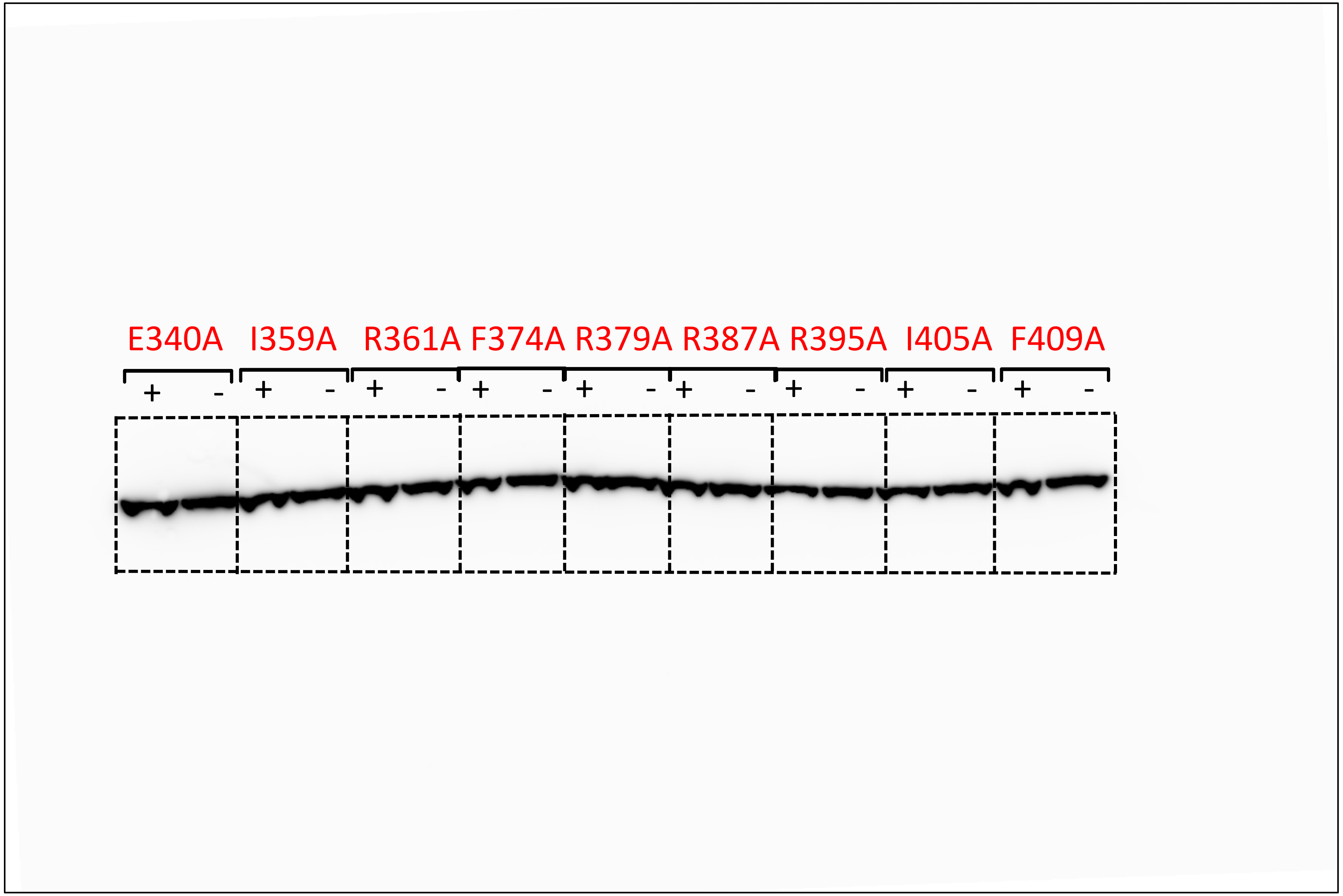

Supplementary Figure 9C

α-DnaA antibody

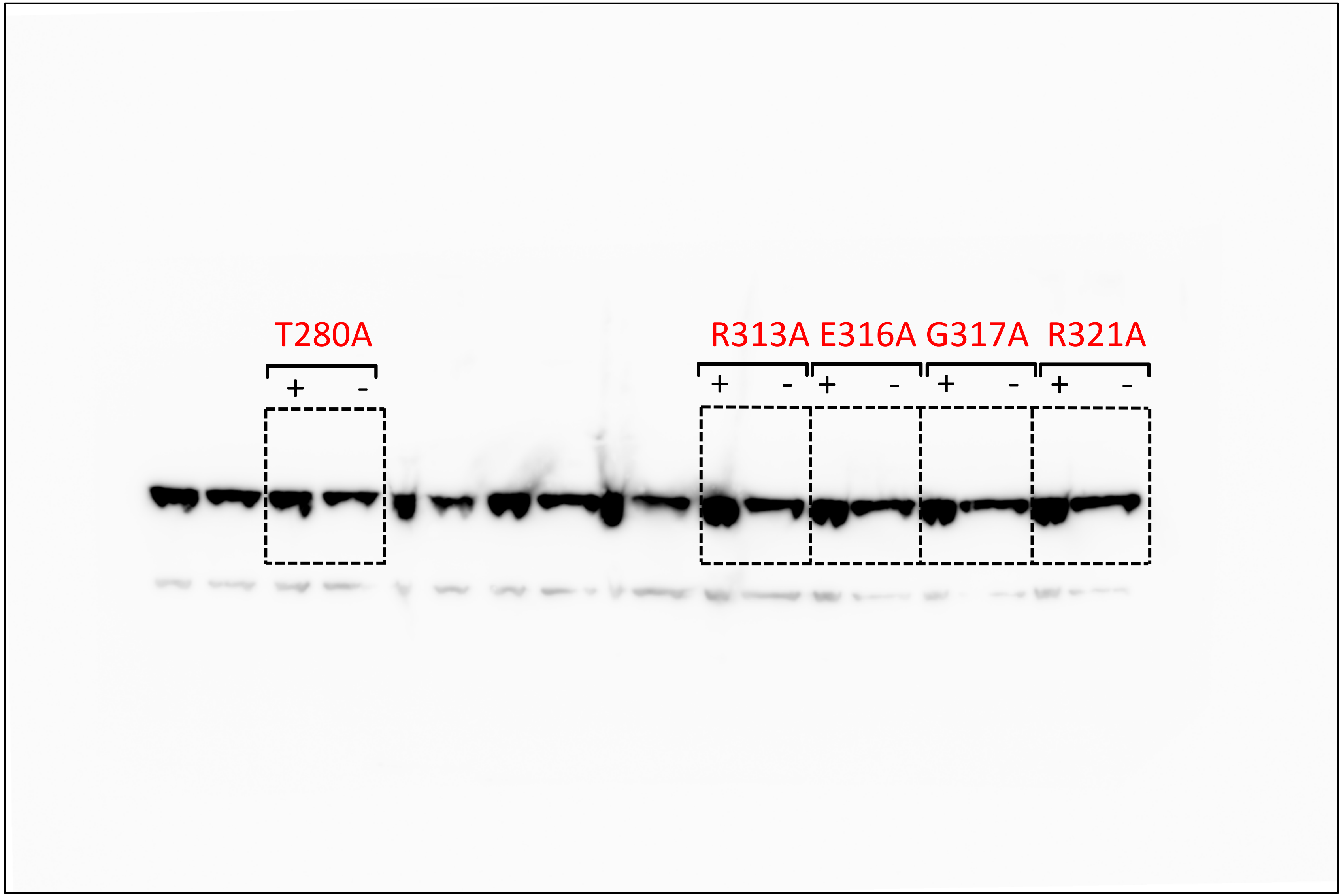

Supplementary Figure 9C

α-FtsZ antibody

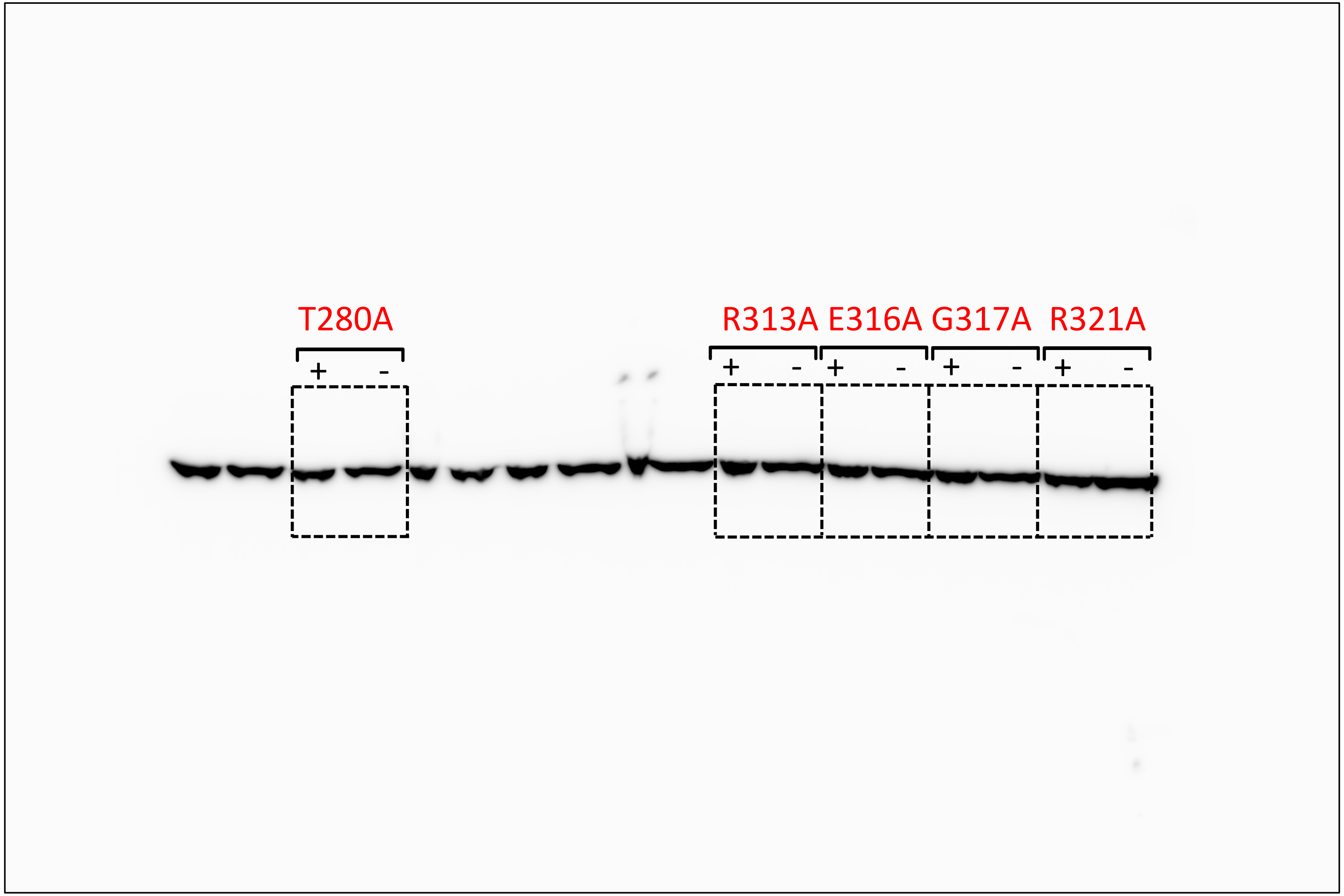

Supplementary Figure 9C

α-DnaA antibody

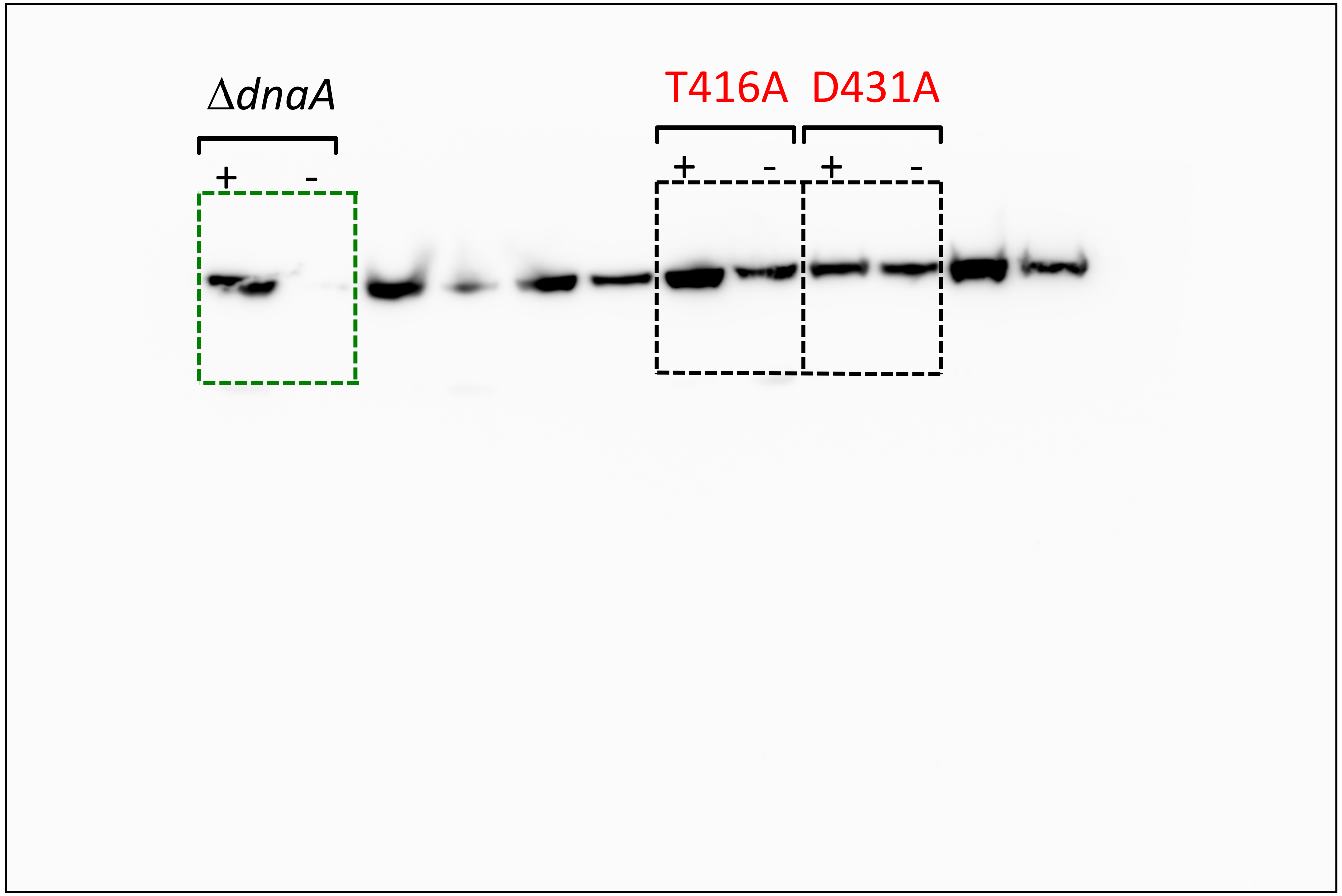

Supplementary Figure 9C

α-FtsZ antibody

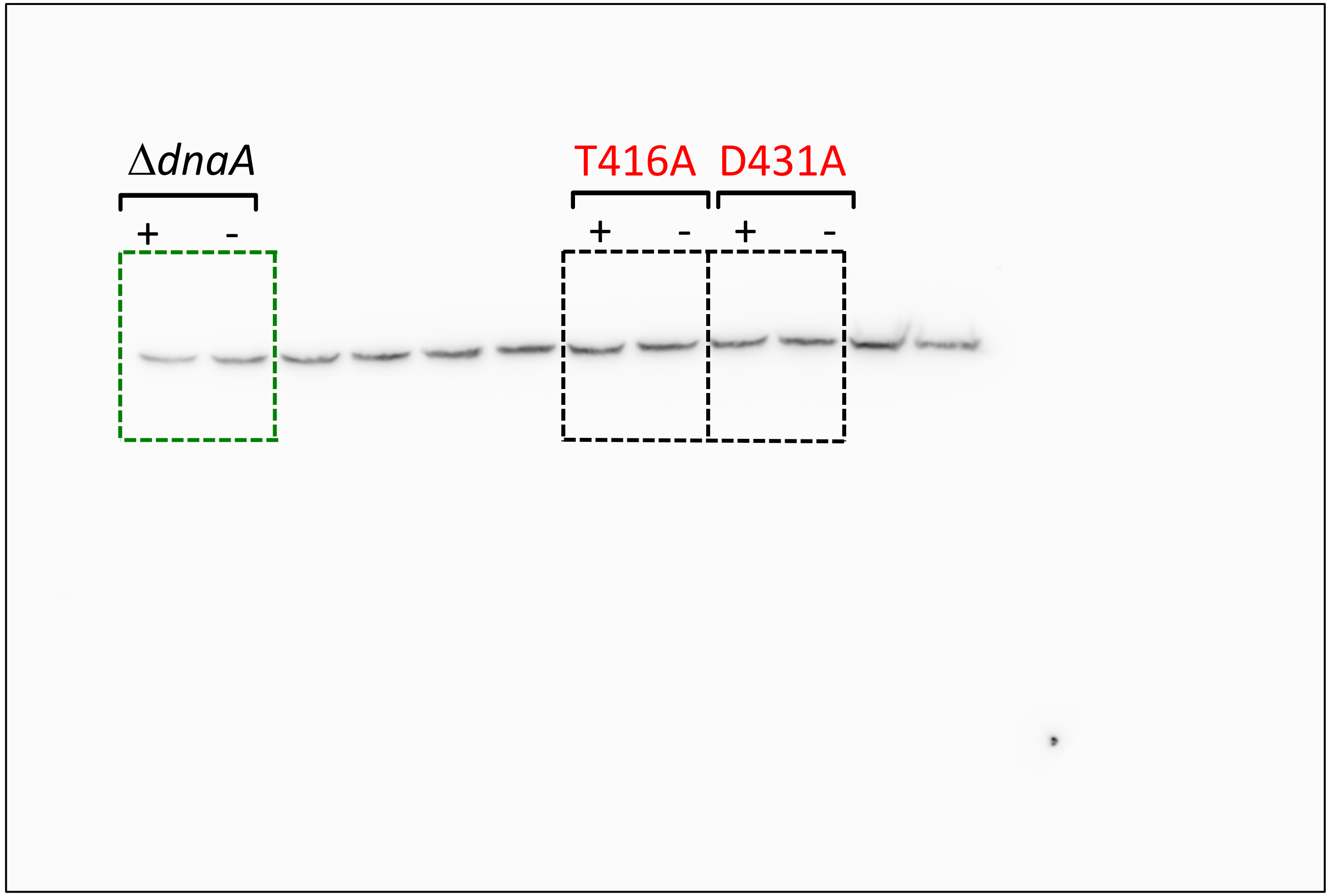

Supplement: Supplementary file 9 — Source Data [file 41467_2023_43823_MOESM9_ESM.zip › Source_Data_File_2_20231024.pdf]
